# Regulation of alternative polyadenylation in the yeast *Saccharomyces cerevisiae* by histone H3K4 and H3K36 methyltransferases

**DOI:** 10.1093/nar/gkaa292

**Published:** 2020-05-01

**Authors:** Katarzyna Kaczmarek Michaels, Salwa Mohd Mostafa, Julia Ruiz Capella, Claire L Moore

**Affiliations:** 1 Department of Developmental, Molecular, and Chemical Biology, Tufts University School of Medicine, Boston, Massachusetts 02111, USA; 2 Tufts University Graduate School of Biomedical Sciences, Boston, MA 02111, USA; 3 Department of Biotechnology, Faculty of Experimental Sciences, Universidad Francisco de Vitoria, Madrid 28223, Spain

## Abstract

Adjusting DNA structure via epigenetic modifications, and altering polyadenylation (pA) sites at which precursor mRNA is cleaved and polyadenylated, allows cells to quickly respond to environmental stress. Since polyadenylation occurs co-transcriptionally, and specific patterns of nucleosome positioning and chromatin modifications correlate with pA site usage, epigenetic factors potentially affect alternative polyadenylation (APA). We report that the histone H3K4 methyltransferase Set1, and the histone H3K36 methyltransferase Set2, control choice of pA site in *Saccharomyces cerevisiae*, a powerful model for studying evolutionarily conserved eukaryotic processes. Deletion of *SET1* or *SET2* causes an increase in serine-2 phosphorylation within the C-terminal domain of RNA polymerase II (RNAP II) and in the recruitment of the cleavage/polyadenylation complex, both of which could cause the observed switch in pA site usage. Chemical inhibition of TOR signaling, which causes nutritional stress, results in Set1- and Set2-dependent APA. In addition, Set1 and Set2 decrease efficiency of using single pA sites, and control nucleosome occupancy around pA sites. Overall, our study suggests that the methyltransferases Set1 and Set2 regulate APA induced by nutritional stress, affect the RNAP II C-terminal domain phosphorylation at Ser2, and control recruitment of the 3′ end processing machinery to the vicinity of pA sites.

## INTRODUCTION

The basic structural unit of chromatin is the nucleosome, consisting of a histone octamer, around which 147 base pairs of DNA are coiled (1,2). Epigenetic modifications provide a higher level of chromatin structure by organizing it into either transcriptionally active euchromatin or inactive heterochromatin. The epigenetic machinery remodels nucleosomes and performs histone posttranslational modifications, which in turn control access of transcription regulatory proteins to DNA, and dictate the initiation and elongation rate of RNA Polymerase II (RNAP II) (3). Epigenetic factors include DNA methyltransferases, histone demethylases, methyltransferases (HMTs), deacetylases (HDACs), acetyltransferases (HATs), dephosphorylases, kinases, deubiquitinases, ubiquitinases and nucleosome remodelers which control nucleosome positioning (3,4). The cell easily modulates its gene expression by dynamic and reversible modifications of chromatin (5–7).

In addition to chromatin remodeling, the cell tunes its transcriptional regulation by switching polyadenylation (pA) sites (8–10). Most human genes have multiple pA sites located not only in their 3′ UTRs, which contain *cis*-regulatory elements, but also within introns and coding sequences. Choice of pA site determines the location at which the pre-mRNA is cleaved and polyadenylated leading to the production of different mRNA isoforms in a process called alternative polyadenylation (APA). Controlling 3′ UTR length may promote or repress mRNA degradation, nuclear export, and translation. Thus, APA leads to production of proteins with different expression levels, structure, function and subcellular localization so that the cell properly responds to different environmental stimuli (11–13). Recent advances have shown that APA and dysregulation of the epigenetic landscape are hallmarks of cancer (4,11,14–18), aging (19–24), DNA damage (8,25,26), differentiation (27–32), metabolism (33–35), heart failure (36,37), pulmonary fibrosis (38,39), neurodegenerative diseases (40,41) and immune responses (28,32,42–46). Due to the lack of knowledge about APA regulation, reversing such dysregulation remains a challenge.

Because polyadenylation occurs co-transcriptionally, epigenetic factors are likely to affect APA. Indeed, slowing the transcriptional elongation rate of RNAP II favors utilization of upstream pA sites (47). Furthermore, specific patterns of nucleosome positioning and chromatin modifications correlate with APA, and are more accurate in predicting functional pA sites than cis elements (48). More specifically, nucleosomes are depleted in the immediate vicinity of pA sites, and preferred pA sites show greater nucleosome occupancy upstream and downstream of the sites, along with RNAP II accumulation upstream of the pA site (48). Moreover, the presence of downstream nucleosomes correlates with the transcriptional termination sites of non-coding RNAs and cryptic transcripts (49). Histone H3K4 (48) and H3K36 (48,50) methylation is strongly associated with pA sites in humans. Methylation of both histone H3K4 and H3K36 has been shown to regulate alternative splicing (51), but it remains unknown whether they regulate APA.

To determine whether histone H3K4 and H3K36 HMTs control APA, we took advantage of *Saccharomyces cerevisiae*, a powerful model for studying evolutionarily conserved eukaryotic processes. In *S. cerevisiae*, Set1 co-transcriptionally methylates histone H3 on lysine 4 (52,53). Histone H3K4me3 is enriched at the 5′ end of genes, histone H3K4me2 in the middle, while histone H3K4me1 is prominent toward the 3′ end of a gene (54,55). Set1 is the HMT in the COMPASS complex, which also includes Bre2, Sdc1, Shg1, Spp1, Swd1, Swd2 and Swd3 (53,56,57). Set1-mediated methylation of histone H3K4 is regulated by the PAF complex (58). Although methylation of histone H3K4 is considered a mark of open chromatin (59–61), it can also repress transcription of rDNA (62), silent mating-type loci (63) and telomeres (63–65), as well as prevent cryptic transcription (63,66–68). Loss of Set1 in *S. cerevisiae* up-regulates the expression of stress responsive genes in a Rad53- and histone H3K4me1-dependent manner (69). Set1 recruits the early termination factor Nrd1, and cells not expressing Set1 and Nrd1 are severely defective for termination of snoRNAs and cryptic unstable transcripts (CUTs) (68). The histone H3K4 demethylase Jhd2 controls choice of pA site by recruiting the 3′ end processing machinery (70) However, the effects of Set1 on the choice of pA site have not been determined. Like nucleosomes, histone H3K4me1 is depleted around pA sites (48). Mammalian genes utilizing upstream pA sites have high levels of histone H3K4me3 at the upstream site, and histone H3K4me3 levels drop down downstream of the pA site (71).

Histone H3K36 methylation in *S. cerevisiae* is catalyzed solely by Set2 (72), and occurs co-transcriptionally (73–76). Like histone H3K4me1, histone H3K36me3 is enriched toward the 3′ end of genes (54,55,77,78). Histone H3K36 methylation in *S. cerevisiae* is regulated by several factors, such as Spt6 (79), the PAF complex (73,80), and the RNAP II C-terminal domain (CTD) Ser2 kinases Ctk1 (73,76,79,81), and Bur1 (80,82,83). Set2 binds RNAP II phosphorylated at Ser2 and Ser5 of its CTD (84). Like methylation of histone H3K4, methylation of histone H3K36 is considered a mark of open chromatin (85), but it can also repress transcription of certain genes (86), and prevent cryptic (87–89) and intragenic transcription (90) mainly via its interaction with the HDAC Rpd3S (88,89,91–93). The repression of intragenic transcription by the histone H3K36 HMT is also conserved in humans (94). Methylation of histone H3K36 and repression of cryptic transcription requires interaction of Set2 with residues of histones H4, H2A and H3 (95,96). Histone H3K36 demethylases increase RNAP II processivity (97), and cells not expressing Set2 or cells with a mutated histone H3K36 residue have elevated levels of RNAP II at the 3′ ends of genes. Preferred pA sites in human genes have high levels of histone H3K36me3 (71), and histone H3K36me3 levels are significantly higher at pA sites in genes with multiple pA sites compared to genes with a single pA site, suggesting a role for histone H3K36me3 in APA regulation (50). Interestingly, levels of histone H3K36me3 drop gradually downstream of pA sites, and the persistence of this mark may lead to increased pausing of RNAP II, which would give more time for processing at an upstream pA site (98,99).

Studies on mechanisms leading to APA have focused on regulation by changing levels of cleavage/polyadenylation proteins or factors that suppress or enhance recruitment of the cleavage/polyadenylation complex to specific pA sites (100–102). Chromatin structure has been recently shown to control alternative promoter choice and alternative splicing (103,104). Previous studies on APA have only demonstrated a correlation between chromatin modifications and pA site choice, but functional validation was not performed (48–50). To test the hypothesis that epigenetic factors affect alternative pA site usage, we looked at utilization of pA sites in *S. cerevisiae* cells not expressing the Set1 or Set2 HMTs. We demonstrate that deletion of *SET1* or *SET2* leads to changes in pA site choice, and negatively affects APA in response to nutritional stress. Furthermore, using a chromatin immunoprecipitation (ChIP) assay, we show that in *set1Δ* and *set2Δ* cells, the amount of 3′ end processing complex at pA sites preferred in the mutants is increased. In addition, *set1Δ* cells had increased RNAP II CTD Ser2 phosphorylation (Ser2-P) at pA sites, which enhances RNAP II’s association with polyadenylation factors (105). Deletion of *SET1* or *SET2* also increased the 3′ end processing efficiency at genes containing single pA sites.

## MATERIALS AND METHODS

### Yeast strains and culture

The yeast mutants were a generous gift from Dr. Hungjiun Liaw (106) and are listed in Table 1. Cells were grown in YPD medium (1% yeast extract, 2% peptone, and 2% glucose) containing 0.004% DMSO with agitation (220 rpm) at 30°C. To induce nutritional stress, cells were grown in the presence or absence of 10 nM rapamycin (AdipoGen, from a 0.25 mM stock dissolved in DMSO) for 2 h in the dark with agitation (220 rpm) at 30°C. For spot assays, yeast cells were cultured in 5 ml of YPD media for 16 h and then diluted to OD_600_ = 0.5, which is about 5 × 10^6^ cells/ml. Ten-fold serial dilutions were spotted on YPD agar plates containing 2 nM rapamycin or DMSO as a solvent control.

**Table 1.** Yeast strains used in this study

Strains	Genotype and carried plasmids
W303α	MATα leu2-3,112 trp1-1 can1-100 ura3-1 ade2-1 his3-11,15
*set1Δ*	MATα leu2-3,112 trp1-1 can1-100 ura3-1 ade2-1 his3-11,15 set1::KANMX6
*set2Δ*	MATα leu2-3,112 trp1-1 can1-100 ura3-1 ade2-1 his3-11,15 set2::KANMX6
WT H3	MATα leu2-3,112 trp1-1 can1-100 ura3-1 ade2-1 his3-11,15 hht1hhf1::KAN, hht2hhf2::KAN, pRS415-HHF1-HHT1
H3K4R	MATα leu2-3,112 trp1-1 can1-100 ura3-1 ade2-1 his3-11,15 hht1hhf1::KAN, hht2hhf2::KAN, pRS415-HHF1-H3K4R
H3K36R	MATα leu2-3,112 trp1-1 can1-100 ura3-1 ade2-1 his3-11,15 hht1hhf1::KAN, hht2hhf2::KAN, pRS415-HHF1-H3K36R

### Immunoblot analysis

Whole-cell lysates were prepared from exponentially growing cultures, using a modified version of the TCA method (107). Briefly, 10 ml of cells were collected by centrifugation and frozen at −80°C. The cell pellet was resuspended in 0.25 ml of cold 20% TCA, and transferred to a 1.5 ml microfuge tube. The cells were broken by vortexing at the highest speed for 3 min at 4°C with acid-washed glass beads. The cell lysate was transferred to a new microfuge tube, avoiding the glass beads. Two 0.5 ml volumes of cold 5% TCA were used to wash beads, and combined with the lysate. The lysate was mixed and the precipitated protein was collected by centrifugation at 14 000 rpm for 10 min at 4°C. The pellet was washed with cold 100% ethanol. Protein was re-suspended in 40 μl of 1M TrisCl (pH 8.0), and 80 μl of 2× SDS loading buffer (60 mM Tris (pH 6.8), 2% SDS, 10% glycerol, 0.2% bromopheonol blue, 100 mM DTT). Samples were heated for 5 min at 95°C before loading onto a 10% SDS-PAGE gel. Proteins were transferred to a polyvinylidene difluoride membrane by electroblotting. Antibodies used for immunoblotting are listed in Table 2.

**Table 2.** Antibodies used in this study

Specificity	Supplier	Catalog #
Anti-Histone H3	Abcam	Ab1791
Anti-Histone H4	Abcam	Ab10156
Anti-H3K4me1	Abcam	Ab8895
Anti-H3K36me3	Abcam	Ab9050
RNAP II CTD Ser2-P	ChromoTek	3E10
RNAP II pan-CTD (4H8)	Santa Cruz	sc-47701
Rna15	Dr H. Domdey	
Pta1	Dr H. Domdey	
α-tubulin (YOL1/34)	Invitrogen	MA1-80189

### qRT-PCR analysis

Total RNA from exponentially growing wild-type or mutant cells was isolated using the Hot Phenol Method (108) and Heavy Phase Lock Gel tubes (Quantabio), and treated with RQ1 DNase (Promega). DNA-free RNA was subjected to reverse transcription using SuperScript III (Invitrogen), and either anchored oligo(dT)20 primer for the 3′ end analysis, or with random hexamers for pA site read-through determination. The resulting cDNA samples were analyzed using the real-time PCR analysis performed in a 12 μl reaction with 417 nM of 10 μM forward and reverse primers, 5 μl of SYBR Green Supermix (BIO-RAD), 1 μl cDNA and 5 μl of distilled water. The primer sequences are listed in Table 3. The expression of the long mRNA isoform of a given gene was normalized to the expression of total mRNA of that gene, and normalized to wild-type.

**Table 3.** Primers used in this study

Primer name	Primer sequence 5′-3′
Anchored oligo d(T) primer	TTTTTTTTTTTTTTTTTTTTVN
Random hexamer	NNNNNN
ISM1 Total Forward	AGCAAGCGATATCTCGCCAA
ISM1 Total Reverse	GTCCATGACAATCCCAGCCA
ISM1 pA4 Forward	CACCAAGCATCACCTCCCAT
ISM1 pA4 Reverse	ATCCTCTTCGGCTGAGTTGG
FAT1 Total/pA1 Forward	TCACGGTGGTTGCCTTGCGT
FAT1 Total/pA1 Reverse	TGGATGTGCGTGGCTCCTGT
FAT1 pA2 Forward	CAAAGGGTTTGGATGGAAATGACAC
FAT1 pA2 Reverse	TCCCAATCAGCAGCGGTCAAG
MDV1 Total/pA1 Forward	TCACACAGAGCTTCCTAACTTCCA
MDV1 Total/pA1 Reverse	ACCCCAGGCGGTATGAGAAATGA
MDV1 pA2 Forward	TGAGGGTCGTGAAAATGGGGAC
MDV1 pA2 Reverse	TCTTCAAATGGGTTGACTTGATTGC
RPB2 Total Forward	GCCTGTAGAGGGTAGATCGAG
RPB2 Total Reverse	TCAGCCCGCAAATACCACAA
RPB2 pA2 Forward	TCATTTGTGCTGATCTTGCCA
RPB2 pA2 Reverse	TGCTTGAAAGTTCTCTCTGCT
RRD2 Total Forward	GGGAAGAATCCCCAACAAGAGC
RRD2 Total Reverse	ACTGCTCATCTGTGAGAGAGGG
RRD2 pA2 Forward	TCTCCACCAAGAGGCCACATAC
RRD2 pA2 Reverse	AGTAGCCGCAATAGCGCTCG
RAD53 Total/pA1 Forward	ACCAAACCTCAAAAGGCCCCGA
RAD53 Total/pA1 Reverse	AGGGGCAGCATTTTCTATGGGT
RAD53 Long Isoform/Between pAs Forward	AACCCGTCTTATGCCTTCCGGG
RAD53 Long Isoform/Between pAs Reverse	GCCGCCTCCGCCCCTTAATC
PDC1 Forward	GCCAGTCTTCGATGCTCCAC
PDC1 Total Reverse	ATCGCTTATTGCTTAGCGTTGG
PDC1 pA Span Reverse	ACTGTCGGCAACTTCTTGTCTGG
RPP1B Total Forward	ACGCTAAGGCTTTGGAAGGTAAGGA
RPP1B Total Reverse	AACCGAAACCCATGTCGTCGTCAGA
RPP1B pA Span Forward	GACGACGACATGGGTTTCGGT
RPP1B pA Span Reverse	TCGTAGCCCTTTCGTATGGACA
RAD53 Promoter Forward	AGGTAAGAAAGCAGAAAAGGACGG
RAD53 Promoter Reverse	GCGTGGATTGCTGTGTGGGT
RAD53 CDS Forward	TCCTAACGGGCCACTTACCTTT
RAD53 CDS Reverse	GGGCCCTTCATGATATGAGCCTCT
RAD53 End of CDS Forward	GTCGGCTAAGAAGCCGCCAG
RAD53 End of CDS Reverse	CGGGGCCTTTTGAGGTTTGGTC
RAD53 pA2 Forward	AGAAGTTTGGGTAATTCGCTGCT
RAD53 pA2 Reverse	TCTTCCCTTACGTGGTAGGC
GRS2 Total Forward	ATAACGATGGCTTCCCCGCT
GRS2 Total Reverse	ACGTAAAGCCTGCGAGATCC
GRS2 pA2 Forward	ACAACCCTGATGAATCGGACTGGG
GRS2 pA2 Reverse	ACAGGCGACAGTCCAAATGTTGAT
RTG2 Total Forward	AGGGTGGTGTTCGAGAGGGTTC
RTG2 Total Reverse	AATGGAGCATAAGGACGGGACGC
RTG2 pA2 Forward	AGTGCTTCCGTTCGTTCCAGA
RTG2 pA2 Reverse	TGCACGCCAATTTTAACCCTCTCT
18S Forward	GATGCCCTTAGACGTTCTGG
18S Reverse	GGCCTCACTAAGCCATTCAA

### Chromatin immunoprecipitation

ChIPs were performed as described previously (109). Quantitative real-time PCR analysis was carried out using SYBR Green reagents (BioRad) with the primers listed in Table 3.

### Statistical analysis

Statistical analysis was carried out using a Student's *t*-test. A two-tailed distribution was performed using a two sample equal variance test. *P < 0.05, **P < 0.01, ***P < 0.001. A *P* value < 0.05 was considered significant.

## RESULTS

### Set1 and Set2 influence usage of pA sites

**Figure 1.**
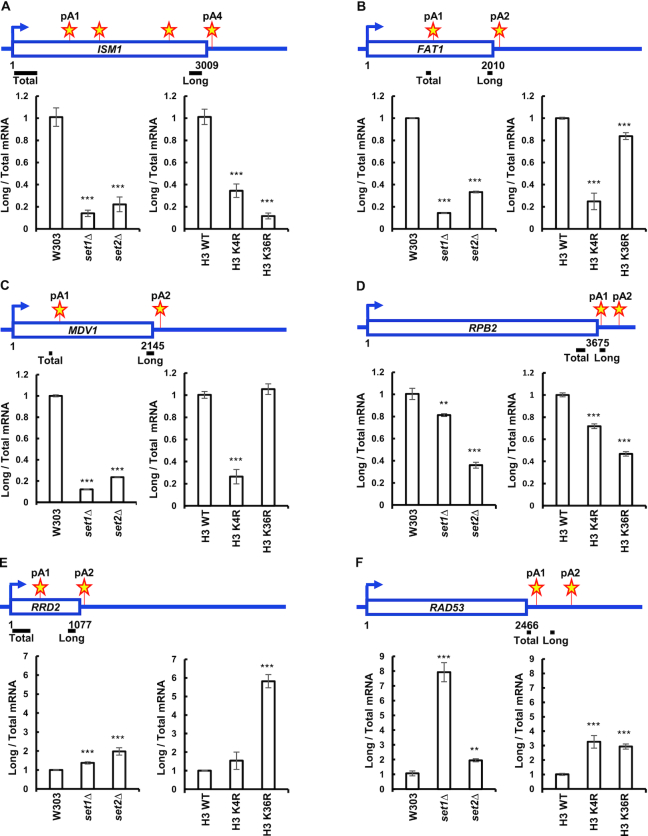
Set1 and Set2 HMTs determine choice of pA site. (**A–F**) Wild-type and *set1Δ*, *set2Δ*, histone H3K4R and histone H3K36R mutants were cultured in YPD media and RNA harvested during exponential growth, followed by reverse transcription using anchored oligo d(T) primers. Total and long gene isoforms of the *ISM1*, *FAT1*, *MDV1*, *RPB2*, *RRD2* and *RAD53* genes were amplified for the 3′ end analysis via qRT-PCR using the primer pairs indicated above the bar graphs. The ratios of long to total mRNAs in the mutant strains were normalized relative to the ratio in the wild-type W303 strain. Stars depict pA sites. Three biological replicates were performed for each gene. Bars show average values ± SD. **P < 0.01, ***P < 0.001 (Student's *t*-test). pA site positions were determined by Graber *et al.* (8).

While histone H3K4 and histone H3K36 methylation correlate with the usage of pA sites (48), it has not been determined whether they influence APA. To assess the role of the histone H3K4 HMT Set1 and the histone H3K36 HMT Set2 in the choice of pA site, we looked at polyadenylation of eight different yeast genes with two or more pA sites. These genes have pA sites located within their open reading frames (*ISM1*, *FAT1*, *MDV1*, *RRD2*, *RTG2*, *GRS2*), or in their 3′ UTRs (*RAD53* and *RPB2*) (Figure 1A–F, Supplementary Figure S1A and B). Switch in pA site usage was measured using total RNA reversely transcribed with oligo d(T) primer to select for polyadenylated RNA. Primers specific for long and total mRNA isoforms were used for qRT-PCR. Long mRNA isoforms were normalized to total mRNA isoforms for a given gene. *set1Δ* and *set2Δ* cells show a strong decrease in use of the *ISM1*, *FAT1*, *MDV1* and *RPB2* downstream pA sites (Figure 1A–D), as measured by the 3′ end amplification assay. Set1 and Set2 had minimal effect on choice of pA site in *GRS2* and *RTG2* (Supplementary Figure S1A and B). The expression of total *ISM1, FAT1* and *MDV1* mRNA was measured with primer sets amplifying regions close to the upstream pA sites and normalized to 18S was increased in *set1Δ* and *set2Δ* cells (Supplementary Figure S2A–C), while that of *RPB2* decreased. In contrast to the other sites that we examined, *set1Δ* and *set2Δ* cells show a strong decrease in use of the *RRD2* and *RAD53* upstream pA sites (Figure 1E and F). Total *RAD53* mRNA normalized to 18S was decreased in *set1Δ* and *set2Δ* cells, while the total *RRD2* mRNA normalized to 18S remained the same (Supplementary Figure S2D–F).

Histone H3K4R and H3K36R mutants, which cannot be methylated by Set1 or Set2, demonstrated similar switches in pA site usage, although to different degrees than *set1Δ* and *set2Δ* cells (Figure 1A–F). Together, these data demonstrate that the absence of Set1 or Set2, as well as mutations in the histone residues that are their targets, change pA site choice, and most often increase use of upstream sites.

A recent paper has reported that the deletion of *SET1* or *SET2* can lead to utilization of internal cryptic promoters (110). Depending on their position relative to our primer sets used to detect total and long mRNA isoforms, activation of these promoters could affect our analysis. Examination of the localization of the internal cryptic promoters reported by Wei *et al.* (110) revealed that there were internal promoters in the *ISM1, MDV1, FAT1* and *RAD53* genes that were significantly increased in *set2Δ* cells, but not in *set1Δ* cells (Supplementary Figure S3A–H). However, activation of the internal cryptic promoters in the *ISM1, MDV1*, and *FAT1* genes would cause the ratio of long to total mRNA isoforms to increase, but we observe a decrease. In summary, other mechanisms, as described below, are likely to be responsible for the APA changes.

### Loss of set1 or Set2 increases processing efficiency at single pA sites

**Figure 2.**
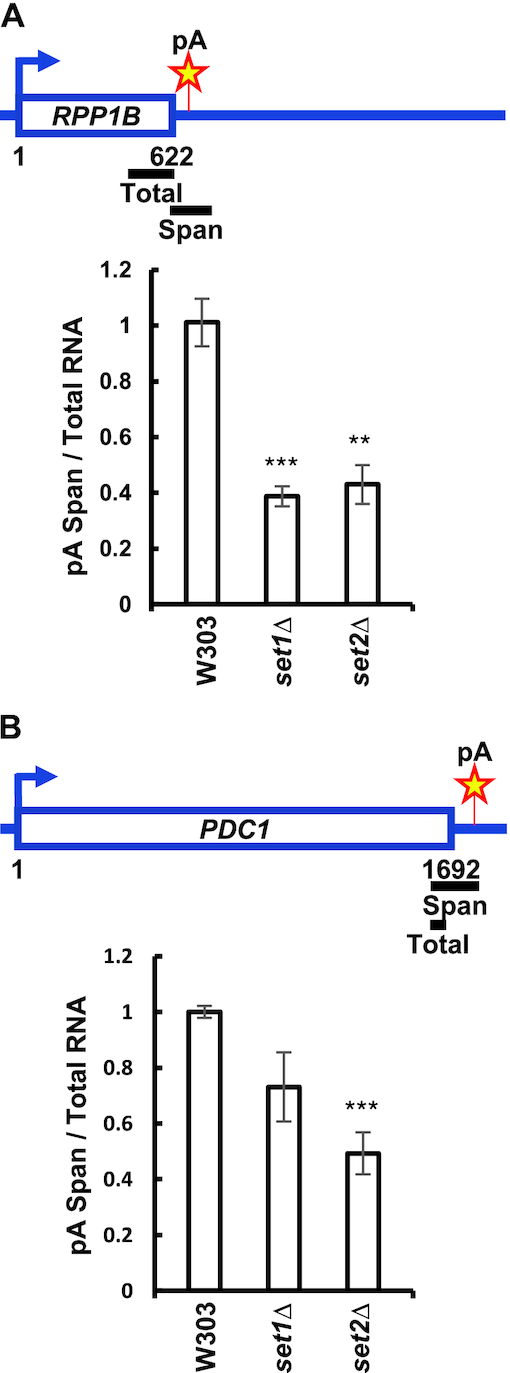
*SET1* and *SET2* deletion enhances utilization of single pA sites. (**A**, **B**) Schematic representation of primer pairs used for qRT-PCR analysis of transcripts reading through the *PDC1* and *RPP1B* pA sites. Total RNA was reversely transcribed using random hexamers. qRT-PCR analysis of RNA was conducted using the primer pairs indicated above the bar graphs to determine the amount of total transcripts and that of transcripts spanning *PDC1* and *RPP1B* pA sites, which represent unprocessed transcripts. The ratios of unprocessed to total transcripts in the mutant strains were normalized relative to the ratio in the wild-type W303 strain. Three biological replicates were performed for each gene. Bars show average values ± SD. **P < 0.01, ***P < 0.001 (Student's *t*-test).

Changes in pA site usage as indicated by the analysis described above can occur if the efficiency of cleavage/polyadenylation is altered. We tested whether Set1 and Set2 alter the efficiency of 3′ end processing in vivo by looking at the level of transcripts that contain sequence upstream and downstream of a pA site. These transcripts represent RNA that has not been processed, and can be detected by RT-qPCR with a primer pair that spans the pA site. We examined the *RPP1B* and *PDC1* genes which have single pA sites. In addition, the genes downstream of *RPP1B* and *PDC1* have the same transcriptional orientation, and are located 496 and 376 bp downstream from the coding sequence of *RPP1B* and *PDC1*, respectively. This gene organization minimizes the possibility of transcriptional interference influencing pA site usage. Absence of Set1 or Set2 decreased the amount of unprocessed RNA, which implies that these chromatin modifiers inhibit processing at the *RPP1B* and *PDC1* pA sites (Figure 2A and B). Total *RPP1B* and *PDC1* mRNA normalized to 18S was decreased in *set1Δ* and *set2Δ* cells, which minimizes the possibility that the decrease in transcripts that span the pA site is due to post-transcriptional stabilization of mature mRNA in these cells (Supplementary Figure S2D–F). Moreover, we were recently able to show a decrease in processing efficiency at single pA sites of *RPP1B* and *PDC1* in the processing-defective *ipa1–1* mutant (111), which further verifies that using these two pA sites we can measure both, an increase and a decrease, in the 3′ end processing. These data indicate that Set1 and Set2 negatively affect the efficiency of cleavage/polyadenylation at these single pA sites.

### Set1 and Set2 control nucleosome occupancy around pA sites

Nucleosome positioning and histone marks are both ways in which the cell alters its chromatin structure. However, only a few studies have addressed the interactions between these dynamic processes. Histone methylation patterns affect nucleosome occupancy (112). For example, cells not expressing Set1 have lower nucleosome occupancy at the *PHO5* promoter (113,114). Likewise, Set2 has been shown to suppress histone exchange over transcribed regions and to suppress histone interactions with histone chaperones (115).

**Figure 3.**
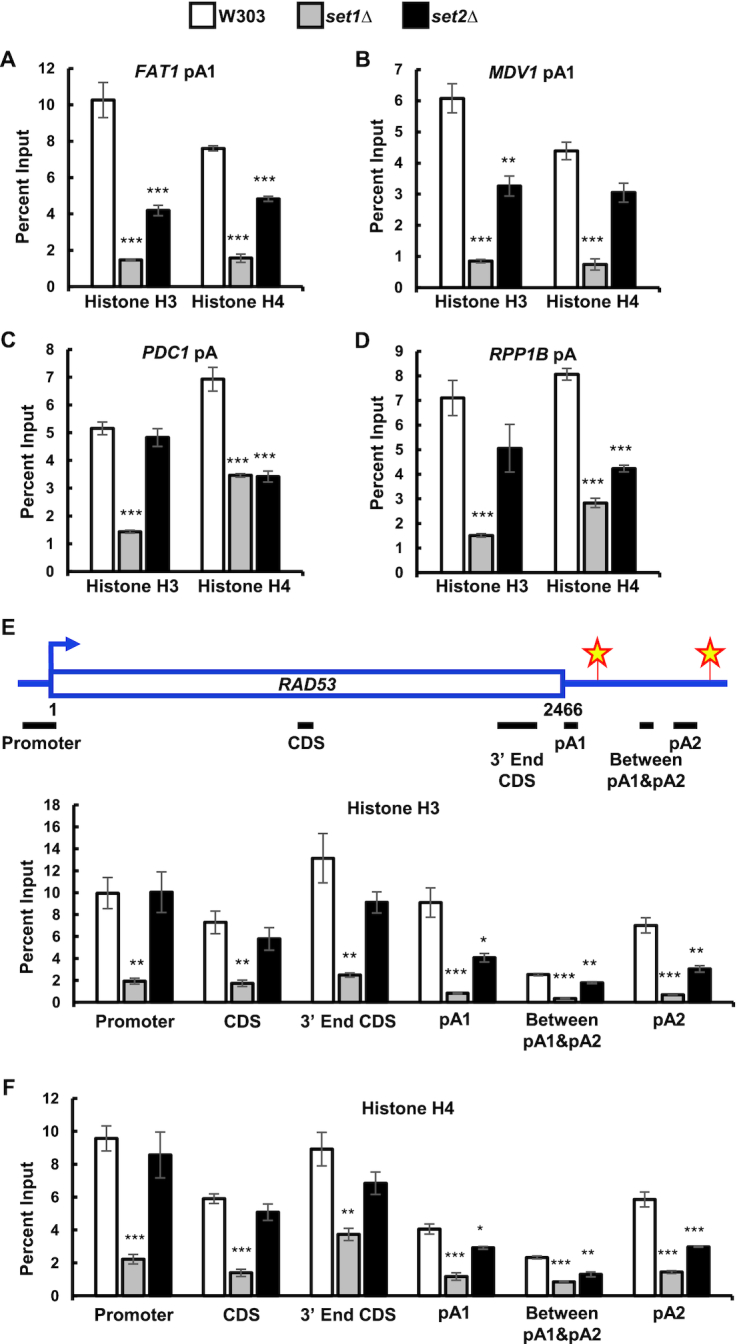
*SET1* and *SET2* deletion decreases nucleosome occupancy around pA sites. (**A–D**) ChIPs for histone H3 and histone H4 around *FAT1*, *MDV1*, *PDC1* and *RPP1B* pA sites in wild-type (W303), *set1Δ* and *set2Δ* cells. (**E**) ChIPs for histone H3, and H4 (**F**) along the *RAD53* gene in wild-type, *set1Δ* and *set2Δ* cells. Primer pairs indicated above the bar graphs were specific for the *RAD53* promoter, coding sequence (CDS), 3′ end of coding sequence, pA1 site, region between pA1 and pA2 sites, as well as pA2 site. Two biological replicates were performed for each gene. Bars show average values ± SD. *P < 0.05, **P < 0.01, ***P < 0.001 (Student's *t*-test).

We examined histone occupancy in *set1Δ* and *set2Δ* cells using antibodies against histone H3 and H4. We focused our analysis on genes with single pA sites, or with well-spaced alternative pA sites to confidently measure changes associated with each pA site via ChIP assays. For this reason, we looked at the pA sites of *FAT1*, *MDV1* and *RAD53*, which have a spacing of at least 500 bp between pA sites. Cells not expressing Set1 have a significant decrease in histone H3 and H4 occupancy around the *FAT1*, *MDV1*, *PDC1*, *RPP1B* and *RAD53* pA sites compared to wild-type cells (Figure 3A–F). Cells not expressing Set2 have decreased histone H3 occupancy around the *FAT1*, *MDV1* and *RAD53* pA sites (Figure 3A, B and E), a moderate decrease at the *RPP1B* pA site, and no change at the *PDC1* site (Figure 3C and D). The *set2Δ* cells also have a strong decrease in histone H4 in the vicinity of the *FAT1*, *PDC1*, *RPP1B* and *RAD53* pA sites and moderate decrease at the *MDV1* pA site (Figure 3A–D, F). Overall, the strongest decrease in nucleosome occupancy is seen in *set1Δ* cells (Figure 3A–F).

**Figure 4.**
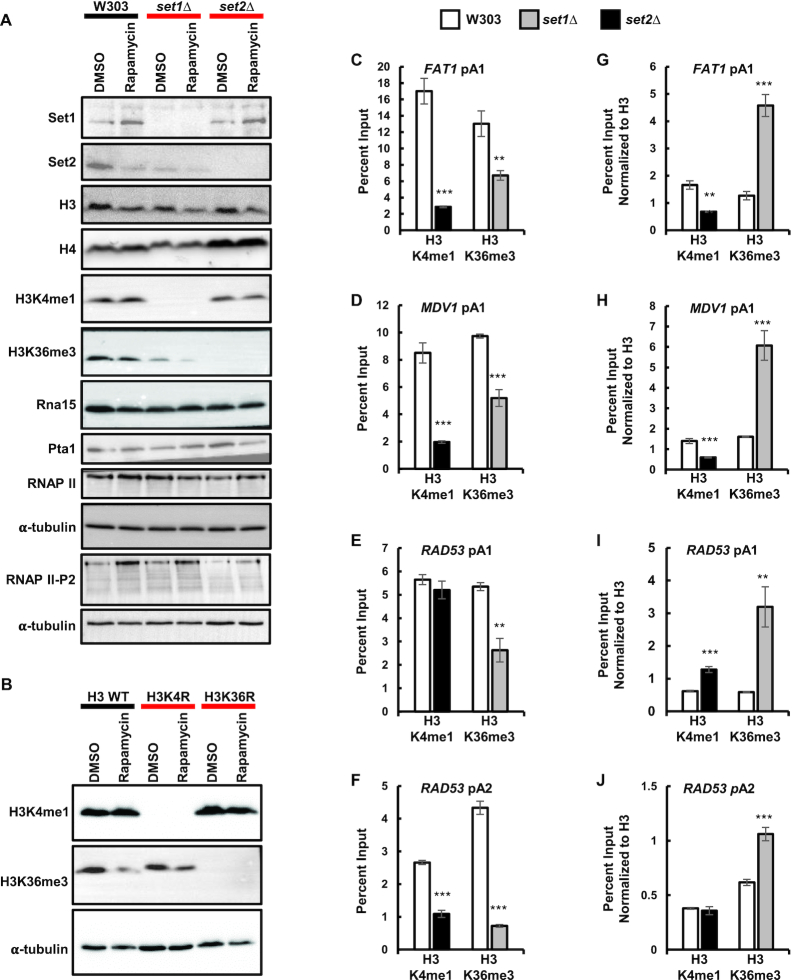
Set1 and Set2 modulate each other. (**A**) Protein levels of Set1, Set2, histone H3, histone H4, H3K4me1, H3K36me3, RNAP II, RNAP II Ser2-P, Rna15 and Pta1 in wild-type, *set1Δ* and *set2Δ* backgrounds. (**B**) Protein levels of histone H3K4me1 and H3K36me3 in wild-type histone H3, histone H3K4R and H3K36R backgrounds. For panel A and B, whole cell extracts from exponentially growing cells in YPD media in the presence of 10 nM rapamycin (for 2 hours) or DMSO were resolved by SDS-PAGE and analyzed by Western blot. α-tubulin was used as a protein loading control. (**C–F**) ChIP of histone H3K4me1 and H3K36me3 to *FAT1*, *MDV1* and *RAD53* pA sites in wild-type, *set1Δ* and *set2Δ* cells. (**G–J**) Same data as in (C–F) normalized to histone H3 ChIP from Figure 3. Two biological replicates were performed for each gene. Bars show average values ± SD. **P < 0.01, ***P < 0.001 (Student's *t*-test).

To see if the loss of histone H3 and H4 in *set1Δ* and *set2Δ* cells is specific to regions near pA sites, we looked at histone H3 and H4 occupancy along the *RAD53* gene. In *set1Δ* cells, histone H3 and H4 levels significantly decreased across the whole gene (Figure 3E and F). *SET2* deletion resulted in a significant loss of histone H3 and H4 occupancy only in the *RAD53* 3′ UTR (Figure 3E and F). The strong decrease in nucleosome occupancy seen in *set1Δ* cells was accompanied by a small decrease in total histone H3 and H4 levels as shown by western blot (Figure 4A). The *set2Δ* cells had unchanged total histone H3 levels, but the histone H4 levels were increased (Figure 4A). These data demonstrate that Set1 and Set2 regulate histone occupancy along genes, especially around pA sites.

### Set1 and Set2 modulate each other

To determine whether Set1 and Set2 influence each other, we looked at histone H3K4me1 in *set2Δ* in cells, and histone H3K36me3 in *set1Δ* cells. Interestingly, the absence of Set1 caused a strong decrease in total histone H3K36me3 levels and presence near pA sites, as measured by western blot and ChIP assay, respectively (Figure 4A, C–F). However, once normalized to total histone H3 levels, *Δset1* cells had increased efficiency of histone H3K36 trimethylation (Figure 4G–J). The absence of Set2 caused a similar decrease in histone H3K4me1 near the *FAT1* and *MDV*1 upstream pA sites, and the *RAD53* downstream pA site, but not the *RAD53* upstream pA site (Figure 4C–F). Once normalized to histone H3 ChIP signal, *set2Δ* cells had decreased monomethylation of histone H3K4 at the *FAT1* and *MDV1* upstream pA sites and decreased monomethylation of histone H3K4 at the downstream *RAD53* pA site, while the H3K4me1 at the *RAD53* upstream pA site remained unchanged (Figure 4G–J). These data suggest possible crosstalk between these different methylation machineries may in turn contribute to the effects of each on pA site choice.

### Set1 and Set2 affect RNAP II Ser2-P and recruitment of cleavage/polyadenylation factors

**Figure 5.**
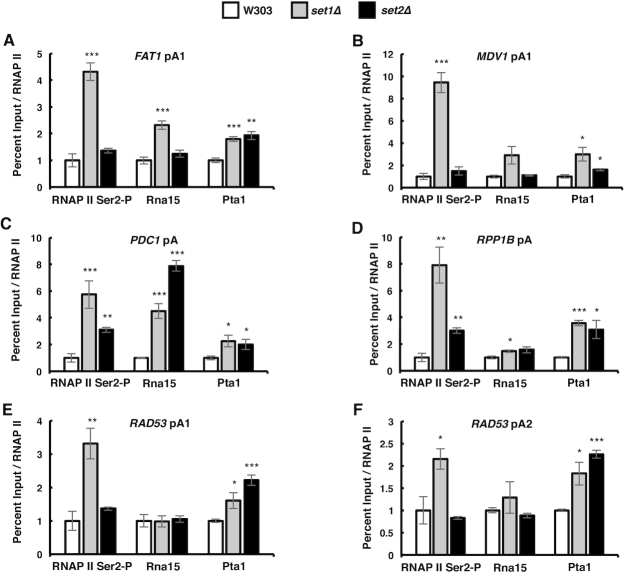
*SET1* and *SET2* deletion enhances the RNAP II CTD Ser2-P and the cleavage/polyadenylation complex levels at pA sites. (**A–F**) ChIP of the RNAP II CTD serine-2 phosphorylation, Rna15 and Pta1 to *FAT1*, *MDV1*, *RAD53*, *PDC1* and *RPP1B* pA sites in wild-type, *set1Δ* and *set2Δ* cells. The values were normalized relative to RNAP II occupancy. Two biological replicates were performed for each gene. Bars show average values ± SD. *P < 0.05, **P < 0.01, ***P < 0.001 (Student's *t*-test).

RNAP II CTD Ser2, Tyr1 and Thr4 phosphorylation is enriched near the 3′ end of genes (116,117). RNAP II CTD Ser2-P is important for the recruitment of the cleavage/polyadenylation complex to the 3′ ends of genes (105). Previous reports showed that cells not expressing Set1 have increased RNAP II CTD Ser5 and Ser7 phosphorylation at the 5′-end of genes (118). We tested whether RNAP II CTD Ser2-P is affected by loss of the Set1 and Set2 HMTs. The amount of RNAP II and RNAP II Ser2-P in total protein extracts was not affected by Set1 depletion, but it was decreased by Set2 depletion (Figure 4A). The *set1Δ* cells had a strong increase in the RNAP II CTD Ser2-P occupancy at all the tested pA sites (Figure 5A–F). Deletion of *SET2* increased RNAP II CTD Ser2-P only at the *PDC1* and RPP1B pA sites, although to a lesser level than that seen in *set1Δ* cells (Figure 5C and D). These data show that the decrease in Set1, and to some degree in Set2, enhances the level of RNAP II CTD Ser2-P near pA sites.

The cleavage/polyadenylation complex in *S. cerevisiae* is composed of Cleavage Factor IA (CF IA), Cleavage Factor IB (CF IB), and holo-CPF, which contains core processing subunits and the Associated with Pta1 (APT) factor (119). To understand the mechanism by which Set1 and Set2 affect pA site choice, we tested whether the HMTs affect recruitment of cleavage/polyadenylation factors to the vicinity of pA sites via ChIP assay. We used the Rna15 subunit of CF IA and the Pta1 subunit of CPF as markers for the two factors, and examined the recruitment of these two proteins to the pA sites of *FAT1*, *MDV1*, *RAD53*, *PDC1* and *RPP1B*. The expression of Rna15 and Pta1 in total protein extracts was not affected by Set1 or Set2 depletion (Figure 4A). However, *set1Δ* cells had increased recruitment of Rna15 to the *FAT1* and *MDV1* upstream pA sites, as well as the *PDC1* and *RPP1B* pA sites (Figure 5A–D), and an increased recruitment of Pta1 to all of the pA sites (Figure 5A–F). The *set2Δ* cells had an increased presence of Rna15 only at the *PDC*1 and *RPP1B* pA sites but an increased recruitment of Pta1 to all of the pA sites (Figure 5A–F). These findings suggest that *SET1* deletion enhances phosphorylation of the RNAP II CTD at Ser2, which in turn leads to increased recruitment of the 3′ end processing factors to the vicinity of pA sites, while *SET2* depletion increases recruitment of the cleavage/polyadenylation complex mostly independent of RNAP II CTD Ser2-P.

### The HMTs set1 and set2 control APA in response to rapamycin

**Figure 6.**
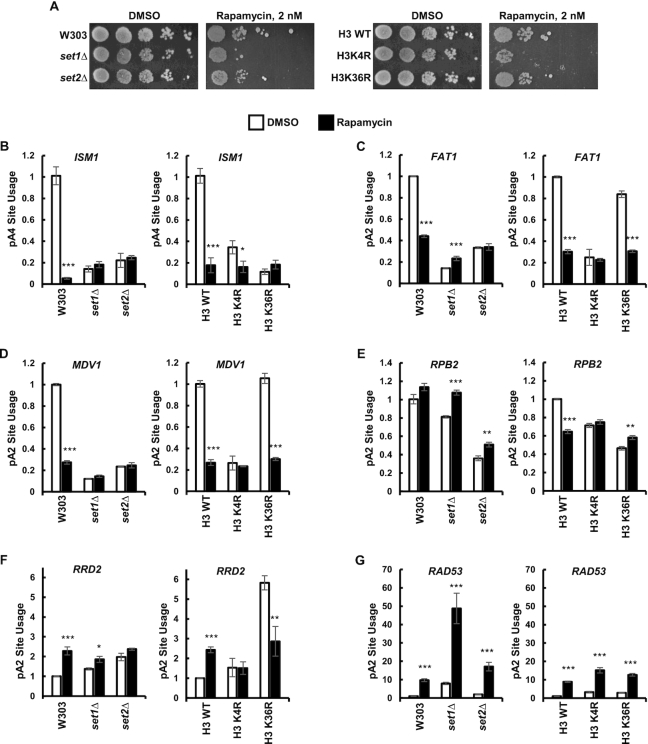
Histone H3K4 and H3K36 methylations are important for resistance to rapamycin. (**A**) *set1Δ*, *set2Δ*, H3K4R and H3K36R cells have increased sensitivity to rapamycin. Ten-fold serial dilutions of indicated strains were spotted on YPD agar containing 2 nM rapamycin or DMSO as a solvent control. (**B–G**) Set1 and Set2 mediate the alternative polyadenylation observed after rapamycin treatment. Wild-type and Set1, Set2, histone H3K4 and histone H3K36 mutants were exponentially grown in YPD media and shifted to media containing 10 nM rapamycin (for 2 h) or DMSO as a solvent control (DMSO data same as in Figure 1). Total RNA was reversely transcribed using anchored oligo d(T) primers. Total and long gene isoforms were amplified via qRT-PCR. The ratios of long to total mRNAs in the mutant strains and in the presence of rapamycin were normalized relative to the ratio in the wild-type W303 strain with DMSO. Three biological replicates were performed for each gene. Bars show average values ± SD. *P < 0.05, **P < 0.01, ***P < 0.001 (Student's *t*-test).

To assess the role of Set1 and Set2 in the switch to alternative pA sites in response to environmental stress, we treated cells with the inhibitor of Target Of Rapamycin (TOR), rapamycin. TOR senses nutrients and regulates cell growth and aging. Mammalian TOR (mTOR) hyperactivation has been shown to lead to global mRNA 3′ UTR shortening in human cells, which upregulates translation of a subset of mRNAs (120). *S. cerevisiae* expresses two TOR proteins: Tor1 and Tor2, both of which are inhibited by rapamycin. Consistent with previous reports (56,121), Set1- and Set2-deficient cells, as well as histone H3K4R and H3K36R mutants, had increased sensitivity to rapamycin-induced nutrient stress (Figure 6A). Wild-type yeast grown for two hours in the presence of rapamycin switched to the upstream pA sites of *ISM1*, *FAT1* and *MDV1* (Figure 6B–D), and to the downstream pA site of *RRD2* and *RAD53* (Figure 6F and G). Rapamycin had minimal effect on *RPB2* APA (Figure 6E).

Cells not expressing Set1 or Set2 were not able to switch to alternative pA sites of *ISM1*, *FAT1*, *MDV1* and *RRD2* (Figure 6B–D, F). Cells expressing histone H3K4R were not able to switch to alternative pA sites of *FAT1*, *MDV1*, *RPB2* and *RRD2* (Figure 6C–F). Cells expressing histone H3K36R were not able to switch to alternative pA sites of *ISM1*, *RPB2* and *RRD2* (Figure 6B, E, F). The *set1Δ* and *set2Δ* cells, as well as mutants of histone H3K4 or H3K36 were still able to switch to the *RAD53* downstream pA site following rapamycin treatment (Figure 6G). Thus, Set1 and Set2 are large contributors to the shift to alternative pA sites in the rapamycin-induced stress condition, at least in part via methylation of histone H3K4 and H3K36 residues.

### Rapamycin alters chromatin structure around pA sites

**Figure 7.**
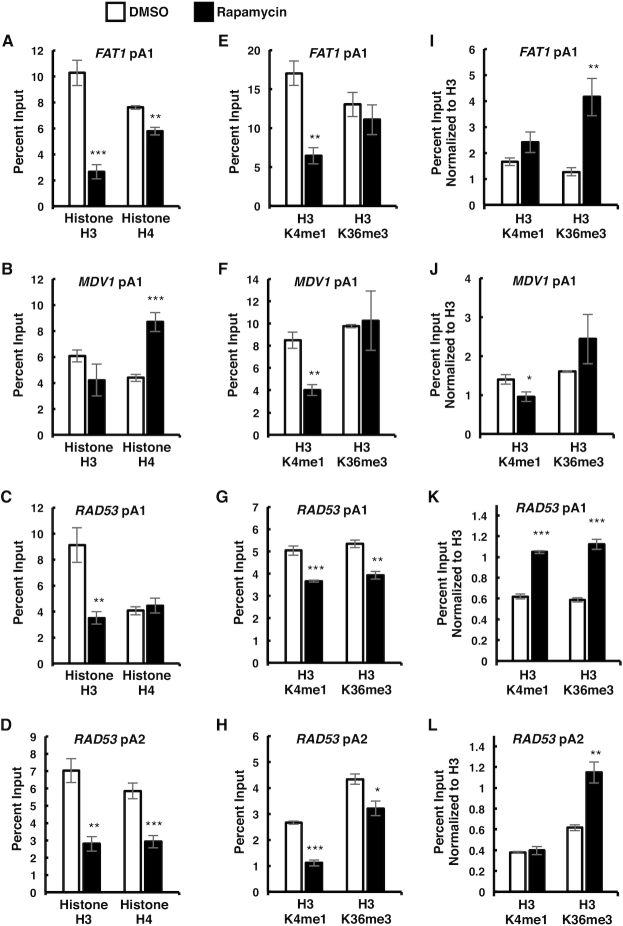
mTOR inhibition leads to epigenetic changes. (**A–D**) ChIPs for histone H3 and histone H4 around *FAT1*, *MDV1*, and *RAD53* pA sites in wild-type *S. cerevisiae* exponentially growing in YPD media and shifted to media containing 10 nM rapamycin (for 2 h) or DMSO as a solvent control. (**E–H**) ChIPs for histone H3K4me1 and histone H3K36me3 near *FAT1*, *MDV1* and *RAD53* pA sites in wild-type cells from (A–D). (**I–L**) Same data as (E–H) normalized to total histone H3 levels from (A–D). Two biological replicates were performed for each gene. Bars show average values ± SD. *P < 0.05, **P < 0.01, ***P < 0.001 (Student's *t*-test).

Epigenetic modifications enable cells to quickly respond to environmental changes. For example, *S. cerevisiae* changes nucleosome occupancy and histone modifications in response to heat shock, osmostress, and different nitrogen conditions (5–7). We investigated whether nutritional stress caused by TOR inhibition affects histone occupancy and methylation within the vicinity of pA sites. We observed a significant decrease in histone H3 levels following rapamycin treatment at the *FAT1* upstream pA site (Figure 7A), and the *RAD53* pA sites (Figure 7C and D). The decrease in histone H3 levels was also detected in whole cell protein extracts via western blot (Figure 4A). Histone H3 levels at the *MDV1* pA site were not as strongly affected by rapamycin treatment (Figure 7B). Histone H4 occupancy around pA sites varied according to the gene, with no change at the *RAD53* upstream pA sites, an increase at the *MDV1* upstream pA site, and a decrease at the *RAD53* downstream pA site, and *FAT1* pA site (Figure 7A–D). The total histone H4 protein levels were not affected by rapamycin treatment (Figure 4A).

Since histone H3K4me1 and H3K36me3 strongly correlate with pA site usage (48), and are found near the 3′ ends of genes (54,55), we examined these histone modifications around pA sites in cells undergoing nutritional stress. Rapamycin treatment decreased histone H3K4me1 around all pA sites (Figure 7E–H), despite Set1 expression being upregulated, and total histone H3K4me1 levels remaining unchanged (Figure 4A). Upon normalization to histone H3 levels, the monomethylation of histone H3K4 was unchanged around the *FAT1* upstream pA site, and the *RAD53* downstream pA site, while it increased around the *RAD53* upstream pA site, and decreased around the *MDV1* upstream pA site (Figure 7I–L). Histone H3K36me3 was decreased around *RAD53* pA sites (Figure 7G and H), but not at the *FAT1* and *MDV1* pA sites (Figure 7E and F). Upon normalization to histone H3 levels, the trimethylation of histone H3K36 was unchanged around the *MDV1* upstream pA site, but it was increased around the *FAT1*, and *RAD53* upstream pA sites, and around the *RAD53* downstream pA site (Figure 7I–L). Set2 expression, and total levels of histone H3K36me3 was downregulated following rapamycin treatment (Figure 4A). The decrease in histone H3K4me1 and H3K36me3 near pA sites can be explained by the overall decrease in nucleosome occupancy around pA sites. The decreased histone H3K36me3 total levels can be also attributed to the decrease in Set2 levels (Figure 4A). Overall, these findings show that rapamycin treatment changes nucleosome occupancy and epigenetic modifications around pA sites, as well as affects the expression of Set1 and Set2.

## DISCUSSION

**Figure 8.**
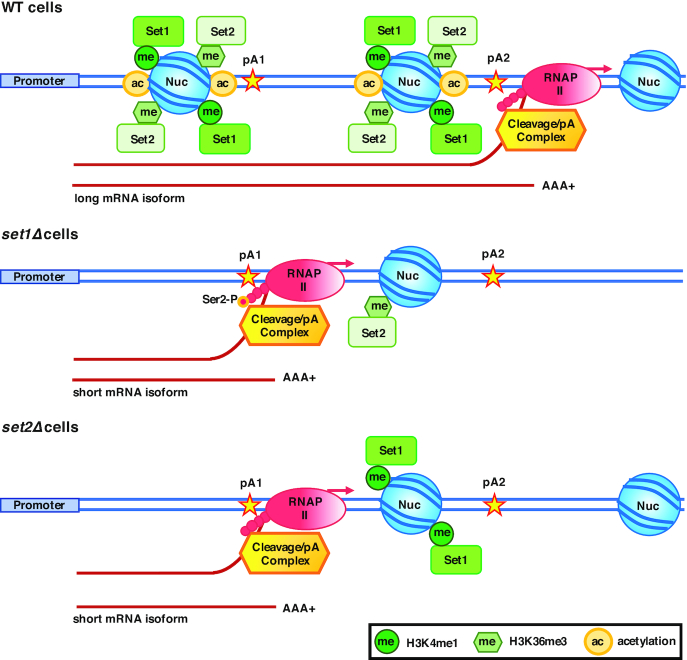
Model for Set1- and Set2-mediated choice of pA site. In wild-type cells, Set1 and Set2 mediate occupancy of histone H3K4me1 and H3K36me3 around pA sites. Methylation of histone H3K4 and H3K36 increases acetylation of nucleosomes, and assures an open chromatin structure, which allows RNAP II transcription to proceed to a downstream pA site (pA2), and production of long mRNA isoforms. Depletion of Set1 leads to loss of histone H3 and H4, reduction of histone H3K36me3, as well as increased phosphorylation of the RNAP II CTD Ser2 near pA1 site, resulting in recruitment of cleavage/polyadenylation factors. Loss of Set2 results in decreased nucleosome (Nuc) occupancy and histone H3K4me1 levels near the pA1 site which in turn enhances recruitment of the 3′ end processing complex.

APA is dysregulated in many human diseases, but despite its relevance to health, the mechanisms regulating it remain a poorly understood aspect of biology. Most studies have looked at the impact of cleavage/polyadenylation factors and RNA-binding proteins on APA. The abundance of histone H3K4me1 and H3K36me3 modifications highly correlate with pA site positions, and they have been proposed to influence pA site selection (48,50). To establish whether these epigenetic processes are responsible for APA regulation, we examined mRNA 3′ end processing in yeast lacking Set1 or Set2 which methylate histone H3K4 and H3K36, respectively, as well as cells with mutations in the histone H3 residues that are targets of these two HMTs. Our findings support a model in which Set1 and Set2 affect RNAP II Ser2-P near pA sites, and hence the recruitment of cleavage/polyadenylation factors, and choice of pA site (Figure 8). H3K4R and H3K36R mutants show similar effects on pA site usage as the *set1Δ* and *set2Δ* mutants, respectively, confirming a role of these histone H3 modifications in APA.

In this study, we show that loss of Set1 or Set2 increases the 3′ end processing efficiency as measured by the decreased accumulation of unprocessed transcripts from genes with single pA sites. By decreasing the 3′ end processing efficiency, the presence of Set1 and Set2 may promote a switch to downstream pA sites, as fewer transcripts cleaved at the upstream pA site would increase the proportion of pre-mRNA processed at the downstream pA site. Indeed, this is the case, as cells lacking Set1 or Set2, or cells with H3K4 or H3H36 mutations, show a switch to upstream pA sites for the majority of genes that we have examined. Furthermore, discrepancies in the pA site utilization between *set1Δ* and H3K4 mutants, and between *set2Δ* and H3K36R mutants, are small, suggesting that Set1 and Set2 affect the choice of pA sites, at least in part, by methylation of their histone targets.

The changes in pA site choice in the absence of Set1 or Set2 could be caused in multiple ways. As summarized in Table 4, we find that loss of these epigenetic factors elicits several changes that could affect pA site usage. These include increased recruitment of the 3′ end processing machinery to transcribed genes, increased phosphorylation of the RNAP II CTD, and alterations in the occupancy of histone H3 and H4 around pA sites. While it is possible that Set1 and Set2 somehow affect the enzymatic activity of the cleavage/polyadenylation complex, such effects have not been reported.

**Table 4.** Summary of key results

		*FAT1*	*MDV1*	*RAD53*	*PDC1*	*RPP1B*
set1Δ cells	Change in pA Site Usage	↑↑↑ pA1	↑↑↑ pA1	↑↑↑ pA2	No Change	↓↓ Read-through
	Histone H3 at pA1 or Single pA	↓↓↓	↓↓↓	↓↓↓	↓↓↓	↓↓↓
	Histone H3 at pA2	ND	ND	↓↓↓	ND	ND
	Histone H4 at pA1 or Single pA	↓↓↓	↓↓↓	↓↓↓	↓↓	↓↓
	Histone H4 at pA2	ND	ND	↓↓↓	ND	ND
	RNAP II Ser2-P at pA1 or Single pA	↑↑↑	↑↑↑	↑↑↑	↑↑↑	↑↑↑
	RNAP II Ser2-P at pA2	ND	ND	↑↑	ND	ND
	Rna15 at pA1 or Single pA	↑↑	↑↑	No Change	↑↑↑	↑
	Pta1 at pA1 or Single pA	↑	↑↑	↑	↑↑	↑↑↑
	Pta1 at pA2	ND	ND	↑	ND	ND
set2Δ cells	Change in pA Site Usage	↑↑↑ pA1	↑↑↑ pA1	↑ pA2	↓↓ Read-through	↓↓ Read-through
	Histone H3 at pA1 or Single pA	↓↓	↓	↓↓	No Change	No Change
	Histone H3 at pA2	ND	ND	↓↓	ND	ND
	Histone H4 at pA1 or Single pA	↓	No Change	No Change	↓↓	↓
	Histone H4 at pA2	ND	ND	↓↓	ND	ND
	RNAP II Ser2-P at pA1 or Single pA	No Change	No Change	No Change	↑↑↑	↑↑↑
	RNAP II Ser2-P at pA2	ND	ND	No Change	ND	ND
	Rna15 at pA1 or Single pA	No Change	No Change	No Change	↑↑↑	↑
	Pta1 at pA1 or Single pA	↑	↑	↑↑	↑↑	↑↑↑
	Pta1 at pA2	ND	ND	↑↑	ND	ND

Not determined (ND).

Phosphorylation of RNAP II CTD Ser2 is required for the recruitment of the 3′ end processing factors (122). In this study, we show that loss of Set1 increases the level of RNAP II CTD Ser2-P at all analyzed pA sites, without affecting its total protein level. *SET2* deletion upregulates RNAP II CTD Ser2-P only at single pA sites, and in contrast to Set1 loss, it decreases total RNAP II, and RNAP II Ser2-P levels. Consistent with the increased RNAP II CTD Ser2-P, *set1Δ* and *set2Δ* mutants have increased recruitment of Rna15 or Pta1 to pA sites, without changes in the overall expression of these subunits of the CF IA and CPF processing factors. However, the extent to which the recruitment of Rna15 and Pta1 to the tested pA sites changes is not always proportional to the increase in RNAP II CTD Ser2-P levels. This difference points to additional mechanisms that control 3′ end machinery recruitment, such as modifications of other RNAP II CTD residues (116,117,123) and interactions of the Rna15-containing CF IA factor with the Spt5 elongation factor and the RNAP II flap loop (124,125). For genes with multiple pA sites, the extent of RNAP II pausing downstream of the pA site and elongation rate of RNAP II between pA sites could also affect the time available for factors to be recruited to the upstream site. This timing could be modulated by changes in chromatin organization and modification.

Both Set1 and Set2 are positioned where they could affect the CTD phosphorylation status, which in turn, could affect recruitment of processing factors to the pA site region. For example, Set1 and Set2 physically interact with RNAP II, and both associate with the newly transcribed RNA (126–128). Set1 gets recruited to RNAP II phosphorylated at Ser5 of its CTD (58,129). However the highest level of Set1 binding to mRNA, as observed by UV crosslinking experiments, occurs right before the pA site, supporting the idea that Set1 will influence 3′ end processing (128). Set2 binds to RNAP II that is phosphorylated at Ser2 and Ser5 of its CTD (84). Set1 and Set2 may regulate RNAP II phosphorylation by controlling the expression, activity or recruitment of the RNAP II CTD kinases or phosphatases. Interestingly, deletion of *SET1* in strains lacking the kinase Ctk1 (Ser2) (130), or RNAP II CTD phosphatases Glc7 (Tyr1) (131) and Rtr1 (Ser5) (118,132–135), and deletion of *SET2* in strains lacking Ctk1 or the Ser2 phosphatase Fcp1 (135,136) alters the cell's fitness, suggesting that the two HMTs and RNAP II CTD modulators functionally interact.

Decreased RNAP II processivity has also been implicated as a mechanism for switching to upstream pA sites (47). It could not only prevent RNAP II from reaching downstream pA sites, but also allow more time for the 3′ end processing machinery to get recruited to, and to work at an upstream pA site. Furthermore, previous studies have indicated that a slow mutant of RNAP II results in increased CTD Ser2-P towards the 5′ of genes (137). Our observations are consistent with such a mechanism, as we find that loss of Set1 or Set2, two HMTs favoring open chromatin (59–61,85), increases RNAP II CTD Ser2-P around upstream, as well as single pA sites. Thus, Set1 and Set2 may also favor the use of downstream pA sites in most of the studied genes because they increase the processivity of RNAP II by altering chromatin structure and indirectly decreasing RNAP II CTD Ser2-P near upstream pA sites. The mechanism by which Set1 and Set2 induce a switch to some upstream pA sites is not clear, and may reflect the fact that these two HMTs promote closed chromatin structure in some genes (62–65,72,86,114,138).

Nucleosome positioning correlates with 3′ end formation (139), and the 3′ ends of genes, especially at pA sites, are depleted of nucleosomes (140). We found that the absence of Set1, and to a smaller degree Set2, decreases histone H3 and H4 occupancy around pA sites. These data are consistent with previous report that cells not expressing the histone H3K4 demethylase Jhd2 have higher histone H3 levels at the *SRG1* 3′ end (141). Likewise, deletion of the histone H3K4 demethylase *KDM5B* results in increased nucleosome occupancy at promoters in embryonic stem cells (142). Methylation of histone H3K4 and H3K36 is likely to affect nucleosome occupancy by affecting nucleosome turnover or remodeling, rather than deposition.

Histone H3K4 and H3K36 residues and HMTs that modify them may also affect pA site choice via interaction with other epigenetic factors. For example, histone H3K36me3 can be bound by NuA3 (143) and NuA4 HAT complexes. Likewise, H3K4me3 recruits HATs such as NuA3 (94), NuA4, SAGA and HBO1. Thus, another way in which a decrease in H3K4 and H3K36 methylation can lead to utilization of upstream pA sites is by decreasing the recruitment of HATs (143), which would then lead to condensed chromatin structure (3,4).

APA and epigenetic modifications allow cells to quickly adjust their RNA and protein composition (8,144,145). For example, nutritional stress results in switch to downstream pA sites in yeast (146), and increases utilization of the downstream *CAT1* pA site, which in turn results in increased expression of the human amino acid transporter Cat1 (147). During cold shock, there is a global switch to upstream pA sites (148), while during heat shock there is a switch to the upstream *HSP70.3* pA site, which results in increased translation of the heat shock protein HSP70.3 (149,150). Stress-induced APA occurs not only in animals and fungi; in plants, hypoxia induces a switch to upstream pA sites (151). The APA response also varies by species. For example, arsenic stress, which causes oxidative stress, leads to utilization of upstream pA sites in mouse (152), while in human cells, it leads to a switch to distal pA sites (9). Likewise, DNA damage in yeast results in a switch to downstream pA sites (8,25), while in human colon carcinoma RKO cells, it favors utilization of upstream pA sites (153).

Set1 and Set2 are important for the cell's proper response to cellular cues and environmental stress. Set2 regulates the proper response to carbon source (154), DNA damage (155–157), and longevity (23,158). Likewise, Set1 is important for the proper response to DNA damage (64,159–161). We have found that during the nutritional stress response induced by the TOR inhibitor rapamycin, several gene transcripts are alternatively polyadenylated. This rapamycin-induced APA correlates with a decrease in histone H3 levels, as well as a decrease in histone H3K4me1 and H3K36me3 around pA sites. Importantly, Set1 and Set2 were required for the rapamycin-induced switch to alternative pA sites. Thus, it is very likely that Set1 and Set2 mediate resistance to rapamycin in part by affecting the choice of pA sites.

Taken together, we present evidence that the epigenetic factors, Set1 and Set2, control choice of pA sites via modulation of RNAP II, and recruitment of the 3′ end processing machinery. As described above, changes in histone H3K4 and H3K36 methylation also affect APA during nutritional stress, and it will be interesting to see if they have similar roles in other cell responses. Set1 and Set2 increase nucleosome occupancy around pA sites, but it remains unknown whether this leads to changes in pA site selection. A better understanding of mechanisms regulating pA site choice, and APA’s role in human health, is required in order to manipulate it to affect disease outcomes.

## Supplementary Material

gkaa292_Supplemental_File

## References

[1] Luger K., Mader A.W., Richmond R.K., Sargent D.F., Richmond T.J. Crystal structure of the nucleosome core particle at 2.8 A resolution. Nature. 1997; 389:251–260. 9305837 10.1038/38444

[2] Richmond T.J., Davey C.A. The structure of DNA in the nucleosome core. Nature. 2003; 423:145–150. 12736678 10.1038/nature01595

[3] Venters B.J., Pugh B.F. How eukaryotic genes are transcribed. Crit. Rev. Biochem. Mol. Biol. 2009; 44:117–141. 19514890 10.1080/10409230902858785 PMC2718758

[4] Miremadi A., Oestergaard M.Z., Pharoah P.D., Caldas C. Cancer genetics of epigenetic genes. Hum. Mol. Genet. 2007; 16:R28–R49. 17613546 10.1093/hmg/ddm021

[5] Shivaswamy S., Iyer V.R. Stress-dependent dynamics of global chromatin remodeling in yeast: dual role for SWI/SNF in the heat shock stress response. Mol. Cell. Biol. 2008; 28:2221–2234. 18212068 10.1128/MCB.01659-07 PMC2268435

[6] Nadal-Ribelles M., Mas G., Millan-Zambrano G., Sole C., Ammerer G., Chavez S., Posas F., de Nadal E. H3K4 monomethylation dictates nucleosome dynamics and chromatin remodeling at stress-responsive genes. Nucleic Acids Res. 2015; 43:4937–4949. 25813039 10.1093/nar/gkv220 PMC4446418

[7] Zhang P., Du G., Zou H., Xie G., Chen J., Shi Z., Zhou J. Genome-wide mapping of nucleosome positions in Saccharomyces cerevisiae in response to different nitrogen conditions. Sci. Rep. 2016; 6:33970. 27659668 10.1038/srep33970 PMC5034280

[8] Graber J.H., Nazeer F.I., Yeh P.C., Kuehner J.N., Borikar S., Hoskinson D., Moore C.L. DNA damage induces targeted, genome-wide variation of poly(A) sites in budding yeast. Genome Res. 2013; 23:1690–1703. 23788651 10.1101/gr.144964.112 PMC3787265

[9] Hollerer I., Curk T., Haase B., Benes V., Hauer C., Neu-Yilik G., Bhuvanagiri M., Hentze M.W., Kulozik A.E. The differential expression of alternatively polyadenylated transcripts is a common stress-induced response mechanism that modulates mammalian mRNA expression in a quantitative and qualitative fashion. RNA. 2016; 22:1441–1453. 27407180 10.1261/rna.055657.115 PMC4986898

[10] Gruber A.J., Zavolan M. Alternative cleavage and polyadenylation in health and disease. Nat. Rev. Genet. 2019; 20:599–614. 31267064 10.1038/s41576-019-0145-z

[11] Mayr C., Bartel D.P. Widespread shortening of 3′UTRs by alternative cleavage and polyadenylation activates oncogenes in cancer cells. Cell. 2009; 138:673–684. 19703394 10.1016/j.cell.2009.06.016 PMC2819821

[12] Tian B., Manley J.L. Alternative polyadenylation of mRNA precursors. Nat Rev Mol Cell Biol. 2016; 18:18–30. 27677860 10.1038/nrm.2016.116 PMC5483950

[13] Batra R., Manchanda M., Swanson M.S. Global insights into alternative polyadenylation regulation. RNA Biol. 2015; 12:597–602. 25892335 10.1080/15476286.2015.1040974 PMC4615881

[14] Akman B.H., Can T., Erson-Bensan A.E. Estrogen-induced upregulation and 3′-UTR shortening of CDC6. Nucleic Acids Res. 2012; 40:10679–10688. 22977174 10.1093/nar/gks855 PMC3510512

[15] Matoulkova E., Sommerova L., Pastorek M., Vojtesek B., Hrstka R. Regulation of AGR2 expression via 3′UTR shortening. Exp Cell Res. 2017; 356:40–47. 28408318 10.1016/j.yexcr.2017.04.011

[16] Ni T.K., Kuperwasser C. Premature polyadenylation of MAGI3 produces a dominantly-acting oncogene in human breast cancer. Elife. 2016; 5:e14730. 27205883 10.7554/eLife.14730 PMC4905742

[17] Miles W.O., Lembo A., Volorio A., Brachtel E., Tian B., Sgroi D., Provero P., Dyson N. Alternative polyadenylation in triple-negative breast tumors allows NRAS and c-JUN to bypass PUMILIO posttranscriptional regulation. Cancer Res. 2016; 76:7231–7241. 27758885 10.1158/0008-5472.CAN-16-0844 PMC5553310

[18] Ye C., Zhou Q., Hong Y., Li Q.Q. Role of alternative polyadenylation dynamics in acute myeloid leukaemia at single-cell resolution. RNA Biol. 2019; 16:785–797. 30810468 10.1080/15476286.2019.1586139 PMC6546370

[19] Mueller A.A., Cheung T.H., Rando T.A. All's well that ends well: alternative polyadenylation and its implications for stem cell biology. Curr. Opin. Cell Biol. 2013; 25:222–232. 23357469 10.1016/j.ceb.2012.12.008 PMC3615088

[20] Shen T., Li H., Song Y., Li L., Lin J., Wei G., Ni T. Alternative polyadenylation dependent function of splicing factor SRSF3 contributes to cellular senescence. Aging (Albany NY). 2019; 11:1356–1388. 30835716 10.18632/aging.101836 PMC6428108

[21] Chen M., Lyu G., Han M., Nie H., Shen T., Chen W., Niu Y., Song Y., Li X., Li H. et al. 3′ UTR lengthening as a novel mechanism in regulating cellular senescence. Genome Res. 2018; 28:285–294. 10.1101/gr.224451.117 PMC5848608 29440281

[22] Mangone M., Manoharan A.P., Thierry-Mieg D., Thierry-Mieg J., Han T., Mackowiak S.D., Mis E., Zegar C., Gutwein M.R., Khivansara V. et al. The landscape of C. elegans 3′UTRs. Science. 2010; 329:432–435. 20522740 10.1126/science.1191244 PMC3142571

[23] Sen P., Dang W., Donahue G., Dai J., Dorsey J., Cao X., Liu W., Cao K., Perry R., Lee J.Y. et al. H3K36 methylation promotes longevity by enhancing transcriptional fidelity. Genes Dev. 2015; 29:1362–1376. 26159996 10.1101/gad.263707.115 PMC4511212

[24] Sen P., Shah P.P., Nativio R., Berger S.L. Epigenetic mechanisms of longevity and aging. Cell. 2016; 166:822–839. 27518561 10.1016/j.cell.2016.07.050 PMC5821249

[25] Yu L., Volkert M.R. UV damage regulates alternative polyadenylation of the RPB2 gene in yeast. Nucleic Acids Res. 2013; 41:3104–3114. 23355614 10.1093/nar/gkt020 PMC3597686

[26] Williamson A.K., Zhu Z., Yuan Z.M. Epigenetic mechanisms behind cellular sensitivity to DNA damage. Cell Stress. 2018; 2:176–180. 31225484 10.15698/cst2018.07.145 PMC6551799

[27] Ogorodnikov A., Levin M., Tattikota S., Tokalov S., Hoque M., Scherzinger D., Marini F., Poetsch A., Binder H., Macher-Goppinger S. et al. Transcriptome 3′end organization by PCF11 links alternative polyadenylation to formation and neuronal differentiation of neuroblastoma. Nat. Commun. 2018; 9:5331. 30552333 10.1038/s41467-018-07580-5 PMC6294251

[28] Qiu F., Fu Y., Lu C., Feng Y., Wang Q., Huo Z., Jia X., Chen C., Chen S., Xu A. Small nuclear ribonucleoprotein polypeptide a-mediated alternative polyadenylation of STAT5B during Th1 cell differentiation. J. Immunol. 2017; 199:3106–3115. 28954886 10.4049/jimmunol.1601872

[29] Spangenberg L., Correa A., Dallagiovanna B., Naya H. Role of alternative polyadenylation during adipogenic differentiation: an in silico approach. PLoS One. 2013; 8:e75578. 24143171 10.1371/journal.pone.0075578 PMC3797115

[30] Grassi E., Santoro R., Umbach A., Grosso A., Oliviero S., Neri F., Conti L., Ala U., Provero P., DiCunto F. et al. Choice of alternative polyadenylation sites, mediated by the RNA-Binding protein elavl3, plays a role in differentiation of inhibitory neuronal progenitors. Front. Cell Neurosci. 2018; 12:518. 30687010 10.3389/fncel.2018.00518 PMC6338052

[31] Brumbaugh J., Di Stefano B., Wang X., Borkent M., Forouzmand E., Clowers K.J., Ji F., Schwarz B.A., Kalocsay M., Elledge S.J. et al. Nudt21 controls cell fate by connecting alternative polyadenylation to chromatin signaling. Cell. 2018; 172:629–631. 29373832 10.1016/j.cell.2017.12.035 PMC5831378

[32] Shulman E.D., Elkon R. Cell-type-specific analysis of alternative polyadenylation using single-cell transcriptomics data. Nucleic. Acids. Res. 2019; 47:10027–10039. 31501864 10.1093/nar/gkz781 PMC6821429

[33] Brutman J.N., Zhou X., Zhang Y., Michal J., Stark B., Jiang Z., Davis J.F. Mapping diet-induced alternative polyadenylation of hypothalamic transcripts in the obese rat. Physiol. Behav. 2018; 188:173–180. 29391168 10.1016/j.physbeh.2018.01.026

[34] Etchegaray J.P., Mostoslavsky R. Interplay between metabolism and epigenetics: a nuclear adaptation to environmental changes. Mol. Cell. 2016; 62:695–711. 27259202 10.1016/j.molcel.2016.05.029 PMC4893201

[35] Sommerkamp P. Altamura S. Altamura S. Renders S., Narr A., Ladel L., Zeisberger P., Eiben P.L., Fawaz M., Rieger M.A., Cabezas-Wallscheid N. et al. Alternative Polyadenylation Landscapes Mediate Hematopoietic Stem Cell Activation and Regulate Glutamine Metabolism. Cell Stem Cell. 2020; 10.1016/j.stem.2020.03.003. 32229311

[36] Creemers E.E., Bawazeer A., Ugalde A.P., van Deutekom H.W., van der Made I., de Groot N.E., Adriaens M.E., Cook S.A., Bezzina C.R., Hubner N. et al. Genome-wide polyadenylation maps reveal dynamic mrna 3′-end formation in the failing human heart. Circ. Res. 2016; 118:433–438. 26671978 10.1161/CIRCRESAHA.115.307082

[37] Gilsbach R., Schwaderer M., Preissl S., Gruning B.A., Kranzhofer D., Schneider P., Nuhrenberg T.G., Mulero-Navarro S., Weichenhan D., Braun C. et al. Distinct epigenetic programs regulate cardiac myocyte development and disease in the human heart in vivo. Nat. Commun. 2018; 9:391. 29374152 10.1038/s41467-017-02762-z PMC5786002

[38] Weng T., Ko J., Masamha C.P., Xia Z., Xiang Y., Chen N.Y., Molina J.G., Collum S., Mertens T.C., Luo F. et al. Cleavage factor 25 deregulation contributes to pulmonary fibrosis through alternative polyadenylation. J. Clin. Invest. 2019; 129:1984–1999. 30830875 10.1172/JCI122106 PMC6486348

[39] Yang I.V., Schwartz D.A. Epigenetics of idiopathic pulmonary fibrosis. Transl Res. 2015; 165:48–60. 24746870 10.1016/j.trsl.2014.03.011 PMC4182166

[40] Patel R., Brophy C., Hickling M., Neve J., Furger A. Alternative cleavage and polyadenylation of genes associated with protein turnover and mitochondrial function are deregulated in Parkinson's, Alzheimer's and ALS disease. BMC Med Genomics. 2019; 12:60. 31072331 10.1186/s12920-019-0509-4 PMC6507032

[41] Berson A., Nativio R., Berger S.L., Bonini N.M. Epigenetic regulation in neurodegenerative diseases. Trends Neurosci. 2018; 41:587–598. 29885742 10.1016/j.tins.2018.05.005 PMC6174532

[42] Allis C.D., Jenuwein T. The molecular hallmarks of epigenetic control. Nat. Rev. Genet. 2016; 17:487–500. 27346641 10.1038/nrg.2016.59

[43] Akman H.B., Erson-Bensan A.E. Alternative polyadenylation and its impact on cellular processes. Microrna. 2014; 3:2–9. 25069507 10.2174/2211536602666131210001152

[44] Singh I., Lee S.H., Sperling A.S., Samur M.K., Tai Y.T., Fulciniti M., Munshi N.C., Mayr C., Leslie C.S. Widespread intronic polyadenylation diversifies immune cell transcriptomes. Nat. Commun. 2018; 9:1716. 29712909 10.1038/s41467-018-04112-z PMC5928244

[45] Jia X., Yuan S., Wang Y., Fu Y., Ge Y., Ge Y., Lan X., Feng Y., Qiu F., Li P. et al. The role of alternative polyadenylation in the antiviral innate immune response. Nat. Commun. 2017; 8:14605. 28233779 10.1038/ncomms14605 PMC5333124

[46] Zhang Q., Cao X. Epigenetic regulation of the innate immune response to infection. Nat. Rev. Immunol. 2019; 19:417–432. 30918351 10.1038/s41577-019-0151-6

[47] Cui Y., Denis C.L. In vivo evidence that defects in the transcriptional elongation factors RPB2, TFIIS, and SPT5 enhance upstream poly(A) site utilization. Mol. Cell. Biol. 2003; 23:7887–7901. 14560031 10.1128/MCB.23.21.7887-7901.2003 PMC207619

[48] Khaladkar M., Smyda M., Hannenhalli S. Epigenomic and RNA structural correlates of polyadenylation. RNA Biol. 2011; 8:529–537. 21508683 10.4161/rna.8.3.15194 PMC3218514

[49] Roy K., Gabunilas J., Gillespie A., Ngo D., Chanfreau G.F. Common genomic elements promote transcriptional and DNA replication roadblocks. Genome Res. 2016; 26:1363–1375. 27540088 10.1101/gr.204776.116 PMC5052057

[50] Lee C.Y., Chen L. Alternative polyadenylation sites reveal distinct chromatin accessibility and histone modification in human cell lines. Bioinformatics. 2013; 29:1713–1717. 23740743 10.1093/bioinformatics/btt288 PMC3702257

[51] Luco R.F., Pan Q., Tominaga K., Blencowe B.J., Pereira-Smith O.M., Misteli T. Regulation of alternative splicing by histone modifications. Science. 2010; 327:996–1000. 20133523 10.1126/science.1184208 PMC2913848

[52] Briggs S.D., Bryk M., Strahl B.D., Cheung W.L., Davie J.K., Dent S.Y., Winston F., Allis C.D. Histone H3 lysine 4 methylation is mediated by Set1 and required for cell growth and rDNA silencing in Saccharomyces cerevisiae. Genes Dev. 2001; 15:3286–3295. 11751634 10.1101/gad.940201 PMC312847

[53] Roguev A., Schaft D., Shevchenko A., Pijnappel W.W., Wilm M., Aasland R., Stewart A.F. The Saccharomyces cerevisiae Set1 complex includes an Ash2 homologue and methylates histone 3 lysine 4. EMBO J. 2001; 20:7137–7148. 11742990 10.1093/emboj/20.24.7137 PMC125774

[54] Li B., Carey M., Workman J.L. The role of chromatin during transcription. Cell. 2007; 128:707–719. 17320508 10.1016/j.cell.2007.01.015

[55] Woo H., Dam Ha S., Lee S.B., Buratowski S., Kim T. Modulation of gene expression dynamics by co-transcriptional histone methylations. Exp. Mol. Med. 2017; 49:e326. 28450734 10.1038/emm.2017.19 PMC6130219

[56] Nagy P.L., Griesenbeck J., Kornberg R.D., Cleary M.L. A trithorax-group complex purified from Saccharomyces cerevisiae is required for methylation of histone H3. Proc. Natl. Acad. Sci. U.S.A. 2002; 99:90–94. 11752412 10.1073/pnas.221596698 PMC117519

[57] Miller T., Krogan N.J., Dover J., Erdjument-Bromage H., Tempst P., Johnston M., Greenblatt J.F., Shilatifard A. COMPASS: a complex of proteins associated with a trithorax-related SET domain protein. Proc. Natl. Acad. Sci. U.S.A. 2001; 98:12902–12907. 11687631 10.1073/pnas.231473398 PMC60797

[58] Krogan N.J., Dover J., Wood A., Schneider J., Heidt J., Boateng M.A., Dean K., Ryan O.W., Golshani A., Johnston M. et al. The Paf1 complex is required for histone H3 methylation by COMPASS and Dot1p: linking transcriptional elongation to histone methylation. Mol. Cell. 2003; 11:721–729. 12667454 10.1016/s1097-2765(03)00091-1

[59] Santos-Rosa H., Schneider R., Bannister A.J., Sherriff J., Bernstein B.E., Emre N.C., Schreiber S.L., Mellor J., Kouzarides T. Active genes are tri-methylated at K4 of histone H3. Nature. 2002; 419:407–411. 12353038 10.1038/nature01080

[60] Bernstein B.E., Humphrey E.L., Erlich R.L., Schneider R., Bouman P., Liu J.S., Kouzarides T., Schreiber S.L. Methylation of histone H3 Lys 4 in coding regions of active genes. Proc. Natl. Acad. Sci. U.S.A. 2002; 99:8695–8700. 12060701 10.1073/pnas.082249499 PMC124361

[61] Boa S., Coert C., Patterton H.G. Saccharomyces cerevisiae Set1p is a methyltransferase specific for lysine 4 of histone H3 and is required for efficient gene expression. Yeast. 2003; 20:827–835. 12845608 10.1002/yea.995

[62] Bryk M., Briggs S.D., Strahl B.D., Curcio M.J., Allis C.D., Winston F. Evidence that Set1, a factor required for methylation of histone H3, regulates rDNA silencing in S. cerevisiae by a Sir2-independent mechanism. Curr. Biol. 2002; 12:165–170. 11818070 10.1016/s0960-9822(01)00652-2

[63] Nislow C., Ray E., Pillus L. SET1, a yeast member of the trithorax family, functions in transcriptional silencing and diverse cellular processes. Mol. Biol. Cell. 1997; 8:2421–2436. 9398665 10.1091/mbc.8.12.2421 PMC25717

[64] Corda Y., Schramke V., Longhese M.P., Smokvina T., Paciotti V., Brevet V., Gilson E., Geli V. Interaction between Set1p and checkpoint protein Mec3p in DNA repair and telomere functions. Nat. Genet. 1999; 21:204–208. 9988274 10.1038/5991

[65] Krogan N.J., Dover J., Khorrami S., Greenblatt J.F., Schneider J., Johnston M., Shilatifard A. COMPASS, a histone H3 (Lysine 4) methyltransferase required for telomeric silencing of gene expression. J. Biol. Chem. 2002; 277:10753–10755. 11805083 10.1074/jbc.C200023200

[66] Kim T., Buratowski S. Dimethylation of H3K4 by Set1 recruits the Set3 histone deacetylase complex to 5′ transcribed regions. Cell. 2009; 137:259–272. 19379692 10.1016/j.cell.2009.02.045 PMC2802783

[67] Pinskaya M., Gourvennec S., Morillon A. H3 lysine 4 di- and tri-methylation deposited by cryptic transcription attenuates promoter activation. EMBO J. 2009; 28:1697–1707. 19407817 10.1038/emboj.2009.108 PMC2699354

[68] Terzi N., Churchman L.S., Vasiljeva L., Weissman J., Buratowski S. H3K4 trimethylation by Set1 promotes efficient termination by the Nrd1-Nab3-Sen1 pathway. Mol. Cell. Biol. 2011; 31:3569–3583. 21709022 10.1128/MCB.05590-11 PMC3165552

[69] Seol J.H., Kim H.J., Yang Y.J., Kim S.T., Youn H.D., Han J.W., Lee H.W., Cho E.J. Different roles of histone H3 lysine 4 methylation in chromatin maintenance. Biochem. Biophys. Res. Commun. 2006; 349:463–470. 16959218 10.1016/j.bbrc.2006.08.122

[70] Blair L.P., Liu Z., Labitigan R.L., Wu L., Zheng D., Xia Z., Pearson E.L., Nazeer F.I., Cao J., Lang S.M. et al. KDM5 lysine demethylases are involved in maintenance of 3′UTR length. Sci. Adv. 2016; 2:e1501662. 28138513 10.1126/sciadv.1501662 PMC5262454

[71] Ji Z., Luo W., Li W., Hoque M., Pan Z., Zhao Y., Tian B. Transcriptional activity regulates alternative cleavage and polyadenylation. Mol. Syst. Biol. 2011; 7:534. 21952137 10.1038/msb.2011.69 PMC3202805

[72] Strahl B.D., Grant P.A., Briggs S.D., Sun Z.W., Bone J.R., Caldwell J.A., Mollah S., Cook R.G., Shabanowitz J., Hunt D.F. et al. Set2 is a nucleosomal histone H3-selective methyltransferase that mediates transcriptional repression. Mol. Cell. Biol. 2002; 22:1298–1306. 11839797 10.1128/mcb.22.5.1298-1306.2002 PMC134702

[73] Krogan N.J., Kim M., Tong A., Golshani A., Cagney G., Canadien V., Richards D.P., Beattie B.K., Emili A., Boone C. et al. Methylation of histone H3 by Set2 in Saccharomyces cerevisiae is linked to transcriptional elongation by RNA polymerase II. Mol. Cell. Biol. 2003; 23:4207–4218. 12773564 10.1128/MCB.23.12.4207-4218.2003 PMC427527

[74] Li B., Howe L., Anderson S., Yates J.R. 3rd, Workman J.L. The Set2 histone methyltransferase functions through the phosphorylated carboxyl-terminal domain of RNA polymerase II. J. Biol. Chem. 2003; 278:8897–8903. 12511561 10.1074/jbc.M212134200

[75] Li J., Moazed D., Gygi S.P. Association of the histone methyltransferase Set2 with RNA polymerase II plays a role in transcription elongation. J. Biol. Chem. 2002; 277:49383–49388. 12381723 10.1074/jbc.M209294200

[76] Xiao T., Hall H., Kizer K.O., Shibata Y., Hall M.C., Borchers C.H., Strahl B.D. Phosphorylation of RNA polymerase II CTD regulates H3 methylation in yeast. Genes Dev. 2003; 17:654–663. 12629047 10.1101/gad.1055503 PMC196010

[77] Barski A., Cuddapah S., Cui K., Roh T.Y., Schones D.E., Wang Z., Wei G., Chepelev I., Zhao K. High-resolution profiling of histone methylations in the human genome. Cell. 2007; 129:823–837. 17512414 10.1016/j.cell.2007.05.009

[78] Nojima T., Gomes T., Grosso A.R., Kimura H., Dye M.J., Dhir S., Carmo-Fonseca M., Proudfoot N.J. Mammalian NET-Seq reveals genome-wide nascent transcription coupled to RNA processing. Cell. 2015; 161:526–540. 25910207 10.1016/j.cell.2015.03.027 PMC4410947

[79] Youdell M.L., Kizer K.O., Kisseleva-Romanova E., Fuchs S.M., Duro E., Strahl B.D., Mellor J. Roles for Ctk1 and Spt6 in regulating the different methylation states of histone H3 lysine 36. Mol. Cell. Biol. 2008; 28:4915–4926. 18541663 10.1128/MCB.00001-08 PMC2519698

[80] Chu Y., Simic R., Warner M.H., Arndt K.M., Prelich G. Regulation of histone modification and cryptic transcription by the Bur1 and Paf1 complexes. EMBO J. 2007; 26:4646–4656. 17948059 10.1038/sj.emboj.7601887 PMC2080810

[81] Fuchs S.M., Kizer K.O., Braberg H., Krogan N.J., Strahl B.D. RNA polymerase II carboxyl-terminal domain phosphorylation regulates protein stability of the Set2 methyltransferase and histone H3 di- and trimethylation at lysine 36. J. Biol. Chem. 2012; 287:3249–3256. 22157004 10.1074/jbc.M111.273953 PMC3270979

[82] Chu Y., Sutton A., Sternglanz R., Prelich G. The BUR1 cyclin-dependent protein kinase is required for the normal pattern of histone methylation by SET2. Mol. Cell. Biol. 2006; 26:3029–3038. 16581778 10.1128/MCB.26.8.3029-3038.2006 PMC1446943

[83] Qiu H., Hu C., Hinnebusch A.G. Phosphorylation of the Pol II CTD by KIN28 enhances BUR1/BUR2 recruitment and Ser2 CTD phosphorylation near promoters. Mol. Cell. 2009; 33:752–762. 19328068 10.1016/j.molcel.2009.02.018 PMC2683426

[84] Kizer K.O., Phatnani H.P., Shibata Y., Hall H., Greenleaf A.L., Strahl B.D. A novel domain in Set2 mediates RNA polymerase II interaction and couples histone H3 K36 methylation with transcript elongation. Mol. Cell. Biol. 2005; 25:3305–3316. 15798214 10.1128/MCB.25.8.3305-3316.2005 PMC1069628

[85] Schaft D., Roguev A., Kotovic K.M., Shevchenko A., Sarov M., Shevchenko A., Neugebauer K.M., Stewart A.F. The histone 3 lysine 36 methyltransferase, SET2, is involved in transcriptional elongation. Nucleic Acids Res. 2003; 31:2475–2482. 12736296 10.1093/nar/gkg372 PMC156053

[86] Landry J., Sutton A., Hesman T., Min J., Xu R.M., Johnston M., Sternglanz R. Set2-catalyzed methylation of histone H3 represses basal expression of GAL4 in Saccharomyces cerevisiae. Mol. Cell. Biol. 2003; 23:5972–5978. 12917322 10.1128/MCB.23.17.5972-5978.2003 PMC180946

[87] Li B., Jackson J., Simon M.D., Fleharty B., Gogol M., Seidel C., Workman J.L., Shilatifard A. Histone H3 lysine 36 dimethylation (H3K36me2) is sufficient to recruit the Rpd3s histone deacetylase complex and to repress spurious transcription. J. Biol. Chem. 2009; 284:7970–7976. 19155214 10.1074/jbc.M808220200 PMC2658090

[88] Joshi A.A., Struhl K. Eaf3 chromodomain interaction with methylated H3-K36 links histone deacetylation to Pol II elongation. Mol. Cell. 2005; 20:971–978. 16364921 10.1016/j.molcel.2005.11.021

[89] Keogh M.C., Kurdistani S.K., Morris S.A., Ahn S.H., Podolny V., Collins S.R., Schuldiner M., Chin K., Punna T., Thompson N.J. et al. Cotranscriptional set2 methylation of histone H3 lysine 36 recruits a repressive Rpd3 complex. Cell. 2005; 123:593–605. 16286008 10.1016/j.cell.2005.10.025

[90] Venkatesh S., Li H., Gogol M.M., Workman J.L. Selective suppression of antisense transcription by Set2-mediated H3K36 methylation. Nat. Commun. 2016; 7:13610. 27892455 10.1038/ncomms13610 PMC5133703

[91] Carrozza M.J., Li B., Florens L., Suganuma T., Swanson S.K., Lee K.K., Shia W.J., Anderson S., Yates J., Washburn M.P. et al. Histone H3 methylation by Set2 directs deacetylation of coding regions by Rpd3S to suppress spurious intragenic transcription. Cell. 2005; 123:581–592. 16286007 10.1016/j.cell.2005.10.023

[92] Li B., Gogol M., Carey M., Pattenden S.G., Seidel C., Workman J.L. Infrequently transcribed long genes depend on the Set2/Rpd3S pathway for accurate transcription. Genes Dev. 2007; 21:1422–1430. 17545470 10.1101/gad.1539307 PMC1877753

[93] Li B., Gogol M., Carey M., Lee D., Seidel C., Workman J.L. Combined action of PHD and chromo domains directs the Rpd3S HDAC to transcribed chromatin. Science. 2007; 316:1050–1054. 17510366 10.1126/science.1139004

[94] Carvalho S., Raposo A.C., Martins F.B., Grosso A.R., Sridhara S.C., Rino J., Carmo-Fonseca M., de Almeida S.F. Histone methyltransferase SETD2 coordinates FACT recruitment with nucleosome dynamics during transcription. Nucleic Acids Res. 2013; 41:2881–2893. 23325844 10.1093/nar/gks1472 PMC3597667

[95] Du H.N., Fingerman I.M., Briggs S.D. Histone H3 K36 methylation is mediated by a trans-histone methylation pathway involving an interaction between Set2 and histone H4. Genes Dev. 2008; 22:2786–2798. 18923077 10.1101/gad.1700008 PMC2569878

[96] Du H.N., Briggs S.D. A nucleosome surface formed by histone H4, H2A, and H3 residues is needed for proper histone H3 Lys36 methylation, histone acetylation, and repression of cryptic transcription. J. Biol. Chem. 2010; 285:11704–11713. 20139424 10.1074/jbc.M109.085043 PMC2857045

[97] Kim T., Buratowski S. Two Saccharomyces cerevisiae JmjC domain proteins demethylate histone H3 Lys36 in transcribed regions to promote elongation. J. Biol. Chem. 2007; 282:20827–20835. 17525156 10.1074/jbc.M703034200

[98] Lian Z., Karpikov A., Lian J., Mahajan M.C., Hartman S., Gerstein M., Snyder M., Weissman S.M. A genomic analysis of RNA polymerase II modification and chromatin architecture related to 3′ end RNA polyadenylation. Genome Res. 2008; 18:1224–1237. 18487515 10.1101/gr.075804.107 PMC2493437

[99] Khaladkar M., Smyda M., Hannenhalli S. Epigenomic and RNA structural correlates of polyadenylation. RNA Biol. 2014; 8:529–537. 10.4161/rna.8.3.15194 PMC3218514 21508683

[100] Bucheli M.E., He X., Kaplan C.D., Moore C.L., Buratowski S. Polyadenylation site choice in yeast is affected by competition between Npl3 and polyadenylation factor CFI. RNA. 2007; 13:1756–1764. 17684230 10.1261/rna.607207 PMC1986811

[101] Gruber A.R., Martin G., Keller W., Zavolan M. Means to an end: mechanisms of alternative polyadenylation of messenger RNA precursors. Wiley Interdiscip. Rev RNA. 2014; 5:183–196. 24243805 10.1002/wrna.1206 PMC4282565

[102] Tian B., Manley J.L. Alternative polyadenylation of mRNA precursors. Nat. Rev. Mol. Cell Biol. 2017; 18:18–30. 27677860 10.1038/nrm.2016.116 PMC5483950

[103] Luco R.F., Allo M., Schor I.E., Kornblihtt A.R., Misteli T. Epigenetics in alternative pre-mRNA splicing. Cell. 2011; 144:16–26. 21215366 10.1016/j.cell.2010.11.056 PMC3038581

[104] Laverriere J.N., L’Hote D., Tabouy L., Schang A.L., Querat B., Cohen-Tannoudji J. Epigenetic regulation of alternative promoters and enhancers in progenitor, immature, and mature gonadotrope cell lines. Mol. Cell. Endocrinol. 2016; 434:250–265. 27402603 10.1016/j.mce.2016.07.010

[105] Ahn S.H., Kim M., Buratowski S. Phosphorylation of serine 2 within the RNA polymerase II C-terminal domain couples transcription and 3′ end processing. Mol. Cell. 2004; 13:67–76. 14731395 10.1016/s1097-2765(03)00492-1

[106] Su W.P., Hsu S.H., Chia L.C., Lin J.Y., Chang S.B., Jiang Z.D., Lin Y.J., Shih M.Y., Chen Y.C., Chang M.S. et al. Combined interactions of plant homeodomain and chromodomain regulate NuA4 activity at DNA double-strand breaks. Genetics. 2016; 202:77–92. 26564157 10.1534/genetics.115.184432 PMC4701104

[107] Wright A.P., Bruns M., Hartley B.S. Extraction and rapid inactivation of proteins from Saccharomyces cerevisiae by trichloroacetic acid precipitation. Yeast. 1989; 5:51–53. 2648697 10.1002/yea.320050107

[108] Ares M. Isolation of total RNA from yeast cell cultures. Cold Spring Harb. Protoc. 2012; 2012:1082–1086. 23028070 10.1101/pdb.prot071456

[109] Pearson E.L., Graber J.H., Lee S.D., Naggert K.S., Moore C.L. Ipa1 is an RNA polymerase II elongation factor that facilitates termination by maintaining levels of the poly(A) site endonuclease Ysh1. Cell Rep. 2019; 26:1919–1933. 30759400 10.1016/j.celrep.2019.01.051 PMC7236606

[110] Wei W., Hennig B.P., Wang J., Zhang Y., Piazza I., Pareja Sanchez Y., Chabbert C.D., Adjalley S.H., Steinmetz L.M., Pelechano V. Chromatin-sensitive cryptic promoters putatively drive expression of alternative protein isoforms in yeast. Genome Res. 2019; 29:1974–1984. 31740578 10.1101/gr.243378.118 PMC6886497

[111] Lee S.D., Liu H.Y., Graber J.H., Heller-Trulli D., Kaczmarek Michaels K., Cerezo J.F., Moore C.L. Regulation of the Ysh1 endonuclease of the mRNA cleavage/polyadenylation complex by ubiquitin-mediated degradation. RNA Biol. 2020; 17:689–702. 32009536 10.1080/15476286.2020.1724717 PMC7237158

[112] Hainer S.J., Martens J.A. Identification of histone mutants that are defective for transcription-coupled nucleosome occupancy. Mol. Cell. Biol. 2011; 31:3557–3568. 21730290 10.1128/MCB.05195-11 PMC3165560

[113] Wang S.S., Zhou B.O., Zhou J.Q. Histone H3 lysine 4 hypermethylation prevents aberrant nucleosome remodeling at the PHO5 promoter. Mol. Cell Biol. 2011; 31:3171–3181. 21646424 10.1128/MCB.05017-11 PMC3147609

[114] Ramakrishnan S., Pokhrel S., Palani S., Pflueger C., Parnell T.J., Cairns B.R., Bhaskara S., Chandrasekharan M.B. Counteracting H3K4 methylation modulators Set1 and Jhd2 co-regulate chromatin dynamics and gene transcription. Nat. Commun. 2016; 7:11949. 27325136 10.1038/ncomms11949 PMC4919544

[115] Venkatesh S., Smolle M., Li H., Gogol M.M., Saint M., Kumar S., Natarajan K., Workman J.L. Set2 methylation of histone H3 lysine 36 suppresses histone exchange on transcribed genes. Nature. 2012; 489:452–455. 22914091 10.1038/nature11326

[116] Kecman T., Heo D.H., Vasiljeva L. Profiling RNA polymerase II phosphorylation genome-wide in fission yeast. Methods Enzymol. 2018; 612:489–504. 30502955 10.1016/bs.mie.2018.07.009

[117] Mayer A., Heidemann M., Lidschreiber M., Schreieck A., Sun M., Hintermair C., Kremmer E., Eick D., Cramer P. CTD tyrosine phosphorylation impairs termination factor recruitment to RNA polymerase II. Science. 2012; 336:1723–1725. 22745433 10.1126/science.1219651

[118] Soares L.M., Buratowski S. Yeast Swd2 is essential because of antagonism between Set1 histone methyltransferase complex and APT (associated with Pta1) termination factor. J. Biol. Chem. 2012; 287:15219–15231. 22431730 10.1074/jbc.M112.341412 PMC3346109

[119] Kumar A., Clerici M., Muckenfuss L.M., Passmore L.A., Jinek M. Mechanistic insights into mRNA 3′-end processing. Curr. Opin. Struct. Biol. 2019; 59:143–150. 31499460 10.1016/j.sbi.2019.08.001 PMC6900580

[120] Chang J.W., Zhang W., Yeh H.S., de Jong E.P., Jun S., Kim K.H., Bae S.S., Beckman K., Hwang T.H., Kim K.S. et al. mRNA 3′-UTR shortening is a molecular signature of mTORC1 activation. Nat. Commun. 2015; 6:7218. 26074333 10.1038/ncomms8218

[121] McDaniel S.L., Hepperla A.J., Huang J., Dronamraju R., Adams A.T., Kulkarni V.G., Davis I.J., Strahl B.D. H3K36 methylation regulates nutrient stress response in saccharomyces cerevisiae by enforcing transcriptional fidelity. Cell Rep. 2017; 19:2371–2382. 28614721 10.1016/j.celrep.2017.05.057 PMC5528882

[122] Hsin J.P., Manley J.L. The RNA polymerase II CTD coordinates transcription and RNA processing. Genes Dev. 2012; 26:2119–2137. 23028141 10.1101/gad.200303.112 PMC3465734

[123] Yurko N.M., Manley J.L. The RNA polymerase II CTD “orphan” residues: Emerging insights into the functions of Tyr-1, Thr-4, and Ser-7. Transcription. 2018; 9:30–40. 28771071 10.1080/21541264.2017.1338176 PMC5791814

[124] Pearson E., Moore C. The evolutionarily conserved Pol II flap loop contributes to proper transcription termination on short yeast genes. Cell Rep. 2014; 9:821–828. 25437538 10.1016/j.celrep.2014.10.007 PMC4250834

[125] Mayer A., Schreieck A., Lidschreiber M., Leike K., Martin D.E., Cramer P. The spt5 C-terminal region recruits yeast 3′ RNA cleavage factor I. Mol. Cell. Biol. 2012; 32:1321–1331. 22290438 10.1128/MCB.06310-11 PMC3302448

[126] Battaglia S., Lidschreiber M., Baejen C., Torkler P., Vos S.M., Cramer P. RNA-dependent chromatin association of transcription elongation factors and Pol II CTD kinases. Elife. 2017; 6:e25637. 28537551 10.7554/eLife.25637 PMC5457138

[127] Sayou C., Millan-Zambrano G., Santos-Rosa H., Petfalski E., Robson S., Houseley J., Kouzarides T., Tollervey D. RNA binding by histone methyltransferases set1 and Set2. Mol. Cell. Biol. 2017; 37:e00165-17. 28483910 10.1128/MCB.00165-17 PMC5492175

[128] Luciano P., Jeon J., El-Kaoutari A., Challal D., Bonnet A., Barucco M., Candelli T., Jourquin F., Lesage P., Kim J. et al. Binding to RNA regulates Set1 function. Cell Discov. 2017; 3:17040. 29071121 10.1038/celldisc.2017.40 PMC5654745

[129] Ng H.H., Robert F., Young R.A., Struhl K. Targeted recruitment of Set1 histone methylase by elongating Pol II provides a localized mark and memory of recent transcriptional activity. Mol. Cell. 2003; 11:709–719. 12667453 10.1016/s1097-2765(03)00092-3

[130] Patturajan M., Conrad N.K., Bregman D.B., Corden J.L. Yeast carboxyl-terminal domain kinase I positively and negatively regulates RNA polymerase II carboxyl-terminal domain phosphorylation. J. Biol. Chem. 1999; 274:27823–27828. 10488128 10.1074/jbc.274.39.27823

[131] Schreieck A., Easter A.D., Etzold S., Wiederhold K., Lidschreiber M., Cramer P., Passmore L.A. RNA polymerase II termination involves C-terminal-domain tyrosine dephosphorylation by CPF subunit Glc7. Nat. Struct. Mol. Biol. 2014; 21:175–179. 24413056 10.1038/nsmb.2753 PMC3917824

[132] Mosley A.L., Pattenden S.G., Carey M., Venkatesh S., Gilmore J.M., Florens L., Workman J.L., Washburn M.P. Rtr1 is a CTD phosphatase that regulates RNA polymerase II during the transition from serine 5 to serine 2 phosphorylation. Mol. Cell. 2009; 34:168–178. 19394294 10.1016/j.molcel.2009.02.025 PMC2996052

[133] Bandyopadhyay S., Mehta M., Kuo D., Sung M.K., Chuang R., Jaehnig E.J., Bodenmiller B., Licon K., Copeland W., Shales M. et al. Rewiring of genetic networks in response to DNA damage. Science. 2010; 330:1385–1389. 21127252 10.1126/science.1195618 PMC3006187

[134] Zhang K., Lin W., Latham J.A., Riefler G.M., Schumacher J.M., Chan C., Tatchell K., Hawke D.H., Kobayashi R., Dent S.Y. The Set1 methyltransferase opposes Ipl1 aurora kinase functions in chromosome segregation. Cell. 2005; 122:723–734. 16143104 10.1016/j.cell.2005.06.021 PMC1794220

[135] Costanzo M., VanderSluis B., Koch E.N., Baryshnikova A., Pons C., Tan G., Wang W., Usaj M., Hanchard J., Lee S.D. et al. A global genetic interaction network maps a wiring diagram of cellular function. Science. 2016; 353:aaf1420. 27708008 10.1126/science.aaf1420 PMC5661885

[136] Gopalakrishnan R., Marr S.K., Kingston R.E., Winston F. A conserved genetic interaction between Spt6 and Set2 regulates H3K36 methylation. Nucleic. Acids. Res. 2019; 47:3888–3903. 30793188 10.1093/nar/gkz119 PMC6486648

[137] Fong N., Saldi T., Sheridan R.M., Cortazar M.A., Bentley D.L. RNA pol II dynamics modulate co-transcriptional chromatin modification, CTD phosphorylation, and transcriptional direction. Mol. Cell. 2017; 66:546–557. 28506463 10.1016/j.molcel.2017.04.016 PMC5488731

[138] Lorenz D.R., Meyer L.F., Grady P.J., Meyer M.M., Cam H.P. Heterochromatin assembly and transcriptome repression by Set1 in coordination with a class II histone deacetylase. Elife. 2014; 3:e04506. 25497836 10.7554/eLife.04506 PMC4383021

[139] Fan X., Moqtaderi Z., Jin Y., Zhang Y., Liu X.S., Struhl K. Nucleosome depletion at yeast terminators is not intrinsic and can occur by a transcriptional mechanism linked to 3′-end formation. Proc. Natl. Acad. Sci. U.S.A. 2010; 107:17945–17950. 20921369 10.1073/pnas.1012674107 PMC2964211

[140] Mavrich T.N., Ioshikhes I.P., Venters B.J., Jiang C., Tomsho L.P., Qi J., Schuster S.C., Albert I., Pugh B.F. A barrier nucleosome model for statistical positioning of nucleosomes throughout the yeast genome. Genome Res. 2008; 18:1073–1083. 18550805 10.1101/gr.078261.108 PMC2493396

[141] Lee K.Y., Chen Z., Jiang R., Meneghini M.D. H3K4 methylation dependent and independent chromatin regulation by JHD2 and SET1 in budding yeast. G3 (Bethesda). 2018; 8:1829–1839. 29599176 10.1534/g3.118.200151 PMC5940172

[142] Kurup J.T., Campeanu I.J., Kidder B.L. Contribution of H3K4 demethylase KDM5B to nucleosome organization in embryonic stem cells revealed by micrococcal nuclease sequencing. Epigenet. Chromatin. 2019; 12:20. 10.1186/s13072-019-0266-9 PMC6444878 30940185

[143] Martin B.J., McBurney K.L., Maltby V.E., Jensen K.N., Brind’Amour J., Howe L.J. Histone H3K4 and H3K36 Methylation Independently Recruit the NuA3 Histone Acetyltransferase in Saccharomyces cerevisiae. Genetics. 2017; 205:1113–1123. 28108585 10.1534/genetics.116.199422 PMC5340327

[144] Fabrizio P., Garvis S., Palladino F. Histone methylation and memory of environmental stress. Cells. 2019; 8:339. 10.3390/cells8040339 PMC6523599 30974922

[145] Xue Y., Acar M. Mechanisms for the epigenetic inheritance of stress response in single cells. Curr. Genet. 2018; 64:1221–1228. 29846762 10.1007/s00294-018-0849-1 PMC6215725

[146] Liu X., Hoque M., Larochelle M., Lemay J.F., Yurko N., Manley J.L., Bachand F., Tian B. Comparative analysis of alternative polyadenylation in S. cerevisiae and S. pombe. Genome Res. 2017; 27:1685–1695. 28916539 10.1101/gr.222331.117 PMC5630032

[147] Aulak K.S., Mishra R., Zhou L., Hyatt S.L., de Jonge W., Lamers W., Snider M., Hatzoglou M. Post-transcriptional regulation of the arginine transporter Cat-1 by amino acid availability. J. Biol. Chem. 1999; 274:30424–30432. 10521420 10.1074/jbc.274.43.30424

[148] Liu Y., Hu W., Murakawa Y., Yin J., Wang G., Landthaler M., Yan J. Cold-induced RNA-binding proteins regulate circadian gene expression by controlling alternative polyadenylation. Sci. Rep. 2013; 3:2054. 23792593 10.1038/srep02054 PMC3690385

[149] Kraynik S.M., Gabanic A., Anthony S.R., Kelley M., Paulding W.R., Roessler A., McGuinness M., Tranter M. The stress-induced heat shock protein 70.3 expression is regulated by a dual-component mechanism involving alternative polyadenylation and HuR. Biochim. Biophys. Acta. 2015; 1849:688–696. 25727182 10.1016/j.bbagrm.2015.02.004

[150] Tranter M., Helsley R.N., Paulding W.R., McGuinness M., Brokamp C., Haar L., Liu Y., Ren X., Jones W.K. Coordinated post-transcriptional regulation of Hsp70.3 gene expression by microRNA and alternative polyadenylation. J. Biol. Chem. 2011; 286:29828–29837. 21757701 10.1074/jbc.M111.221796 PMC3191024

[151] de Lorenzo L., Sorenson R., Bailey-Serres J., Hunt A.G. Noncanonical alternative polyadenylation contributes to gene regulation in response to hypoxia. Plant Cell. 2017; 29:1262–1277. 28559476 10.1105/tpc.16.00746 PMC5502444

[152] Zheng D., Wang R., Ding Q., Wang T., Xie B., Wei L., Zhong Z., Tian B. Cellular stress alters 3′UTR landscape through alternative polyadenylation and isoform-specific degradation. Nat. Commun. 2018; 9:2268. 29891946 10.1038/s41467-018-04730-7 PMC5995920

[153] Devany E., Park J.Y., Murphy M.R., Zakusilo G., Baquero J., Zhang X., Hoque M., Tian B., Kleiman F.E. Intronic cleavage and polyadenylation regulates gene expression during DNA damage response through U1 snRNA. Cell Discov. 2016; 2:16013. 27462460 10.1038/celldisc.2016.13 PMC4906801

[154] Kim J.H., Lee B.B., Oh Y.M., Zhu C., Steinmetz L.M., Lee Y., Kim W.K., Lee S.B., Buratowski S., Kim T. Modulation of mRNA and lncRNA expression dynamics by the Set2-Rpd3S pathway. Nat. Commun. 2016; 7:13534. 27892458 10.1038/ncomms13534 PMC5133700

[155] Carvalho S., Vitor A.C., Sridhara S.C., Martins F.B., Raposo A.C., Desterro J.M., Ferreira J., de Almeida S.F. SETD2 is required for DNA double-strand break repair and activation of the p53-mediated checkpoint. Elife. 2014; 3:e02482. 24843002 10.7554/eLife.02482 PMC4038841

[156] Jha D.K., Strahl B.D. An RNA polymerase II-coupled function for histone H3K36 methylation in checkpoint activation and DSB repair. Nat. Commun. 2014; 5:3965. 24910128 10.1038/ncomms4965 PMC4052371

[157] Li F., Mao G., Tong D., Huang J., Gu L., Yang W., Li G.M. The histone mark H3K36me3 regulates human DNA mismatch repair through its interaction with MutSalpha. Cell. 2013; 153:590–600. 23622243 10.1016/j.cell.2013.03.025 PMC3641580

[158] Ryu H.Y., Rhie B.H., Ahn S.H. Loss of the Set2 histone methyltransferase increases cellular lifespan in yeast cells. Biochem. Biophys. Res. Commun. 2014; 446:113–118. 24607280 10.1016/j.bbrc.2014.02.061

[159] Schramke V., Neecke H., Brevet V., Corda Y., Lucchini G., Longhese M.P., Gilson E., Geli V. The set1Delta mutation unveils a novel signaling pathway relayed by the Rad53-dependent hyperphosphorylation of replication protein A that leads to transcriptional activation of repair genes. Genes Dev. 2001; 15:1845–1858. 11459833 10.1101/gad.193901 PMC312739

[160] Kanoh J., Francesconi S., Collura A., Schramke V., Ishikawa F., Baldacci G., Geli V. The fission yeast spSet1p is a histone H3-K4 methyltransferase that functions in telomere maintenance and DNA repair in an ATM kinase Rad3-dependent pathway. J. Mol. Biol. 2003; 326:1081–1094. 12589755 10.1016/s0022-2836(03)00030-5

[161] Faucher D., Wellinger R.J. Methylated H3K4, a transcription-associated histone modification, is involved in the DNA damage response pathway. PLoS Genet. 2010; 6:e1001082. 20865123 10.1371/journal.pgen.1001082 PMC2928815

