# Supplementary material for: Regulation of alternative polyadenylation in the yeast Saccharomyces cerevisiae by histone H3K4 and H3K36 methyltransferases
Source: Nucleic Acids Res. 2020 May 1;48(10):5407–25. doi: 10.1093/nar/gkaa292 (PMC7261179; doi:10.1093/nar/gkaa292)

SUPPLEMENTARY FIGURES

SUPPLEMENTARY FIGURE 1. **Set1 and Set2 do not determine choice of the *GRS2* and *RTG2* pA sites.** (A-B) Wild-type, *set1Δ*, and *set2Δ* cells were cultured in YPD media and RNA harvested during exponential growth, followed by reverse transcription using anchored oligo d(T) primers. Total and long gene isoforms of the *GRS2*, and *RTG2* genes were amplified for the 3'-end analysis via qRT-PCR using the primer pairs indicated above the bar graphs. Long mRNA isoforms were normalized to total mRNA isoforms for a given gene. Stars depict pA sites. Three biological replicates were performed for each gene. Bars show average values ± SD. pA site positions were determined by J.H. Graber et al. (8).

SUPPLEMENTARY FIGURE 2. **Set1 and Set2 affect mRNA levels in a gene-specific fashion.** (A-H) Wild-type, *set1Δ*, and *set2Δ* cells were cultured in YPD media and RNA harvested during exponential growth, followed by reverse transcription using anchored oligo d(T) primers. Total and long gene isoforms of the *ISM1*, *FAT1*, *MDV1*, *RPB2*, *RRD2*, *RAD53*, *PDC1*, and *RPP1B* genes were measured via qRT-PCR, and normalized to 18S mRNA. Three biological replicates were performed for each gene. Bars show average.

SUPPLEMENTARY FIGURE 3. ***SET2* deletion activates internal cryptic promoters.** (A-H) Internal cryptic promoters in the *ISM1*, *FAT1*, *MDV1*, *RPB2*, *RRD2*, *RAD53*, *PDC1*, and *RPP1B* genes that were significantly induced in *set2Δ* cells. No internal cryptic promoters were significantly changed in the studied genes in *set1Δ* cells. Internal cryptic promoters' positions in *set1Δ* and *set2Δ* cells were determined by W. Wei et al. (110).

Supplementary Figure 1

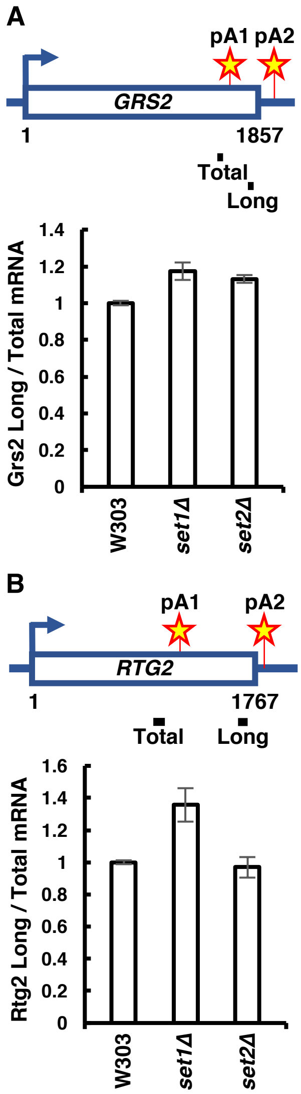

Supplementary Figure 2

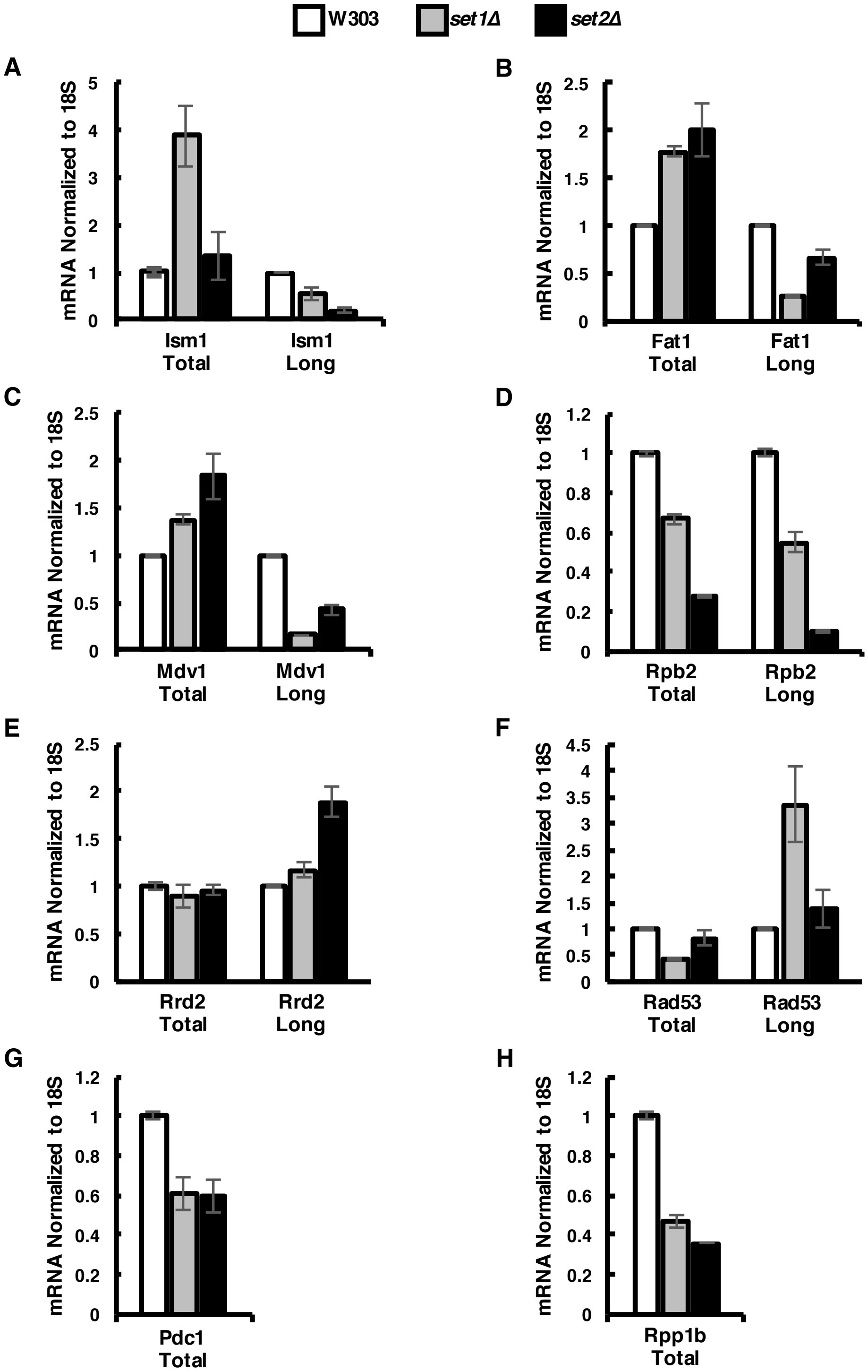

Supplementary Figure 3

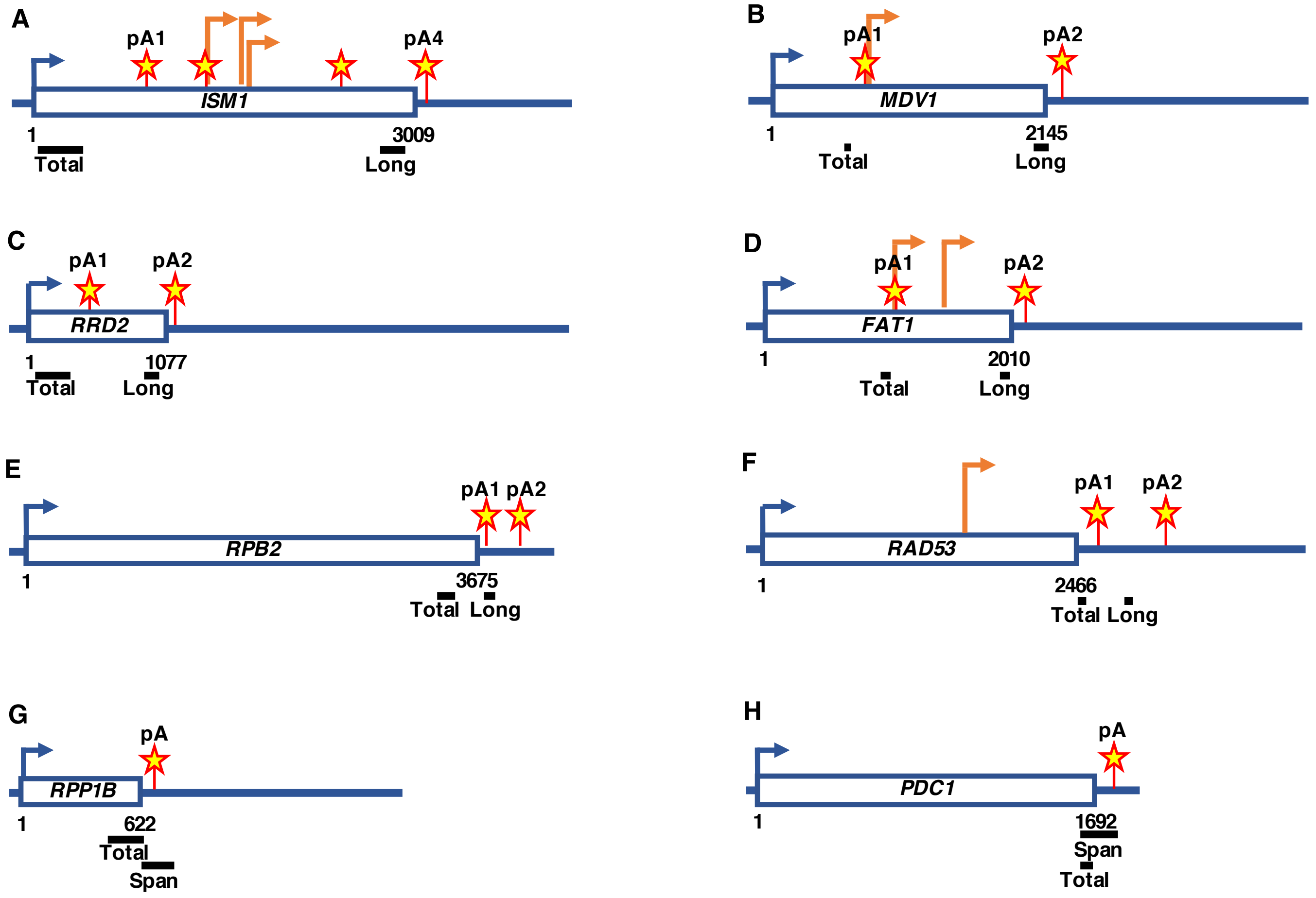

Supplement: gkaa292_Supplemental_File [file gkaa292_supplemental_file.pdf]
